# PICH promotes sister chromatid disjunction and co-operates with topoisomerase II in mitosis

**DOI:** 10.1038/ncomms9962

**Published:** 2015-12-08

**Authors:** Christian F. Nielsen, Diana Huttner, Anna H. Bizard, Seiki Hirano, Tian-Neng Li, Timea Palmai-Pallag, Victoria A. Bjerregaard, Ying Liu, Erich A. Nigg, Lily Hui-Ching Wang, Ian D. Hickson

**Affiliations:** 1 Center for Chromosome Stability and Center for Healthy Aging, Department of Cellular and Molecular Medicine, University of Copenhagen, DK-2200 Copenhagen, Denmark; 2 Center for Protein Research, University of Copenhagen, DK-2200 Copenhagen, Denmark; 3 Weatherall Institute of Molecular Medicine, John Radcliffe Hospital, University of Oxford, OX3 9DS Oxford, UK; 4 Institute of Molecular and Cellular Biology & Department of Medical Science, National Tsing Hua University, 30013 Hsinchu, Taiwan; 5 Biozentrum, University of Basel, CH-4056 Basel, Switzerland

## Abstract

PICH is a SNF2 family DNA translocase that binds to ultra-fine DNA bridges (UFBs) in mitosis. Numerous roles for PICH have been proposed from protein depletion experiments, but a consensus has failed to emerge. Here, we report that deletion of *PICH* in avian cells causes chromosome structural abnormalities, and hypersensitivity to an inhibitor of Topoisomerase II (Topo II), ICRF-193. ICRF-193-treated *PICH*^*−/−*^ cells undergo sister chromatid non-disjunction in anaphase, and frequently abort cytokinesis. PICH co-localizes with Topo IIα on UFBs and at the ribosomal DNA locus, and the timely resolution of both structures depends on the ATPase activity of PICH. Purified PICH protein strongly stimulates the catalytic activity of Topo II *in vitro*. Consistent with this, a human *PICH*^−/−^ cell line exhibits chromosome instability and chromosome condensation and decatenation defects similar to those of ICRF-193-treated cells. We propose that PICH and Topo II cooperate to prevent chromosome missegregation events in mitosis.

## Abstract (web summary)

During mitosis the translocase PICH binds to ultrafine bridges formed from DNA catenanes that are unresolved by topoisomerase II. In this study, the authors show that PICH stimulates toposiomerase II activity and that they cooperate to resolve these structures.

## Graphical abstract

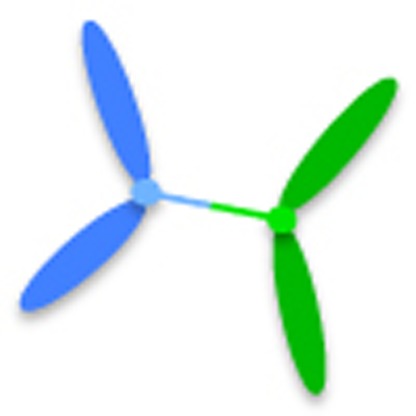

The accurate replication of the genome in S-phase and the faithful segregation of sister chromatids in mitosis are key events required for the maintenance of chromosome stability. During DNA replication, the sister chromatids become topologically connected because of the combined effect of protein-mediated cohesion^1^ and the catenation of the newly replicated sister DNA duplexes^2^. Cohesion is removed from chromosome arms in the mitotic prophase via the action of WAPL protein and mitotic kinases^3^. However, centromeric cohesion is preserved at this stage because of the inhibitory action of shugoshin 1 and protein phosphatase 2A on the cohesin protein complex^4^. This leads to the characteristic appearance of X-shaped metaphase chromosomes held together only at their centromeric region. Persistence of centromeric cohesion is crucial for the development of a stable bipolar attachment of the kinetochores to spindle microtubules^5^. This persistent centromeric cohesion in metaphase has consequences for the structure of the centromeric DNA in that it leads to preservation of DNA catenanes at this specific location. These catenanes can seemingly only be accessed by the mitotic DNA decatenase, topoisomerase II (Topo II), at the metaphase-to-anaphase transition when the centromeric cohesin ring complex is cleaved by separase^6^. Topo II is a double-stranded DNA (dsDNA)-specific decatenase, implying that the majority of the catenanes that persist from S-phase into mitosis at centromeres define fully replicated DNA (reviewed in ref. 7).

A failure to resolve catenanes in a timely fashion can lead to the formation of anaphase DNA bridges. These can be either ‘bulky' chromatinized bridges that can be stained with 4,6-diamidino-2-phenylindole (DAPI) or ultra-fine anaphase bridges (UFBs) that are nucleosome-free and DAPI-negative^8^,^9^. The vast majority of UFBs that arise spontaneously originate from centromeres (denoted as C-UFBs)^8^,^9^. The frequency of C-UFBs is greatly enhanced following exposure of cells to the Topo II inhibitor, ICRF-193, consistent with a role for Topo II in resolving DNA catenations that underlie UFB formation^9^,^10^,^11^. C-UFBs can be distinguished from the fragile site-associated UFBs (FS-UFBs) that form on chromosome arms in response to DNA replication stress, in that FS-UFBs are revealed by the presence of twin FANCD2 protein foci in early mitosis^10^. FS-UFBs most likely represent regions of the genome that are not fully replicated at the time of mitotic entry. Once formed, FS-UFBs and C-UFBs can be visualized by immunofluorescence using antibodies specific for the PICH protein^8^,^9^. PICH is an SNF2 family member that possesses dsDNA translocase activity. PICH also has the unusual property of acting as a form of DNA ‘tension sensor', in that it binds more efficiently *in vitro* to dsDNA that is exposed to stretching forces^12^. This has been proposed to explain why PICH decorates UFBs along their entire length irrespective of the stage of anaphase, as UFBs tethered at each end to the separating sister chromatids would be expected to be under tension because of forces exerted by the mitotic spindle^12^.

A number of studies have sought to identify the effects of disrupting PICH function on chromosome structure and stability. Using RNA interference in human cells, several groups have reported phenotypic abnormalities including mitotic checkpoint failure^8^, disruption of chromosomal architecture in prometaphase^13^,^14^,^15^ and increased chromosome missegregation in anaphase^13^,^14^,^16^,^17^. However, the mitotic checkpoint phenotype has been demonstrated to reflect an off-target effect of the short interfering RNAs used^18^, whereas other phenotypes were found in some, but not in other, studies. Moreover, it is not clear whether the nature and frequency of UFBs are affected in any way by the abrogation of PICH function, because depletion of PICH causes loss of most protein markers that normally allow UFBs to be visualized using immunofluorescence, such as the Bloom's syndrome protein, BLM^9^. However, recent data^19^,^20^ indicate that TOPBP1 localization defines a subset of UFBs that can be visualized in the absence of PICH.

To circumvent these problems, in this study we have generated a vertebrate cell line with complete loss of PICH function via targeted inactivation of the *PICH* gene in avian DT40 cells. We show that these *PICH*^*−/−*^ cells exhibit a number of mitotic defects that are exacerbated by the inhibition of Topo II. In addition, we show that PICH and Topo II co-localize on UFBs and at the rDNA locus in mitosis. To complement these studies, we have also generated a *PICH*^*−/−*^ human cell line, which displays defects in sister chromatid disjunction. These data, coupled with the finding that PICH strongly stimulates the catalytic activity of Topo II *in vitro*, lead us to propose a model in which PICH and Topo II functionally cooperate in mitosis to ensure faithful chromosome segregation, and to prevent chromosome non-disjunction and polyploidization.

## Results

### Generation of chicken DT40 *PICH* ^*−/−*^ cell lines

**Figure 1:**
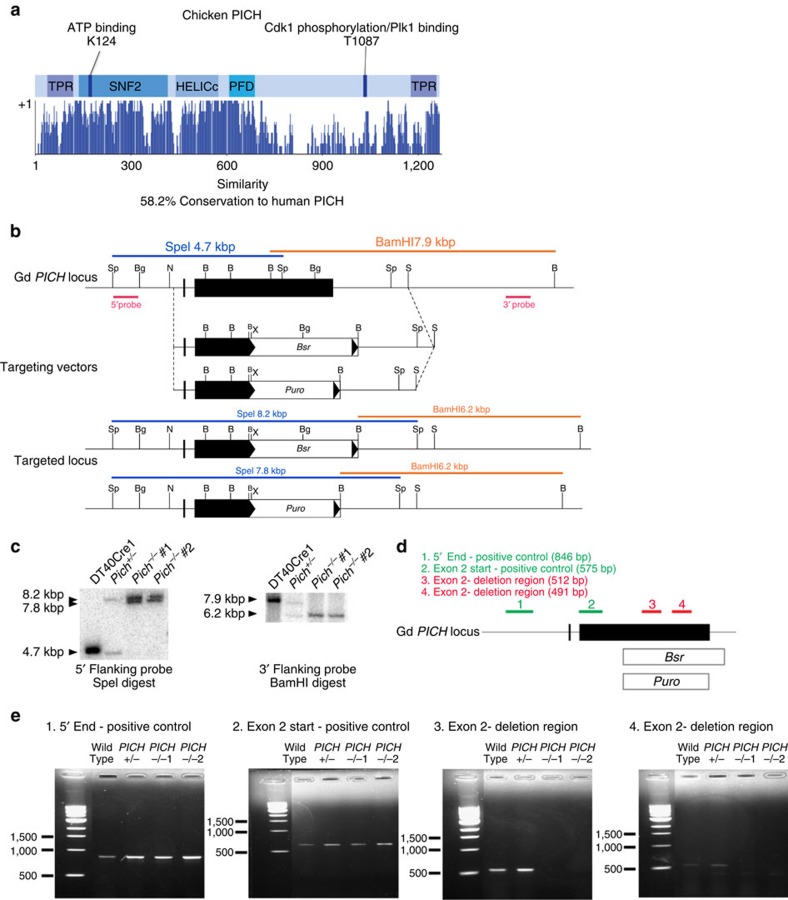
Generation and validation of *PICH*^*−/−*^ cells. (**a**) Conservation of the chicken and human PICH proteins. The defined domains, designated TPR, SNF2, HELICc and PFD, are abbreviations for Tetratricopeptide repeat, sucrose non-fermenting, helicase superfamily c-terminal domain and PICH family domain, respectively. Conservation is defined as the % of amino-acid positions that are identical or from the same functional group, and is depicted as a series of peaks aligned along the PICH sequence. Data were extracted from the NCBI database. (**b**) The *PICH* gene targeting strategy at the chicken *PICH* locus. The black boxes represent the *PICH* exons and the *PICH* homology regions flanking the *Bsr* or *Puro* resistance genes in the targeting vectors. Positions of the 5′ and 3′ validation Southern blotting probes are shown in pink. The size and position of probed DNA that would be expected in an unmodified or a targeted *PICH* locus after digestion with *Spe*I (blue) or *Bam*HI (orange) are shown. (**c**) Southern blots probing the 5′ end and 3′ end of *PICH* to confirm insertion of the *Puro* and *Bsr* genes in genomic DNA digested with either *Spe*1 or *Bam*H1. (**d**) Positions of positive (1–2) and negative (3–4) control primers at the Gd *PICH* locus. (**e**) The gene knockout was validated by PCR with primer sets 1–4 on isolated genomic DNA from cells of the indicated genotypes.

**Figure 2:**
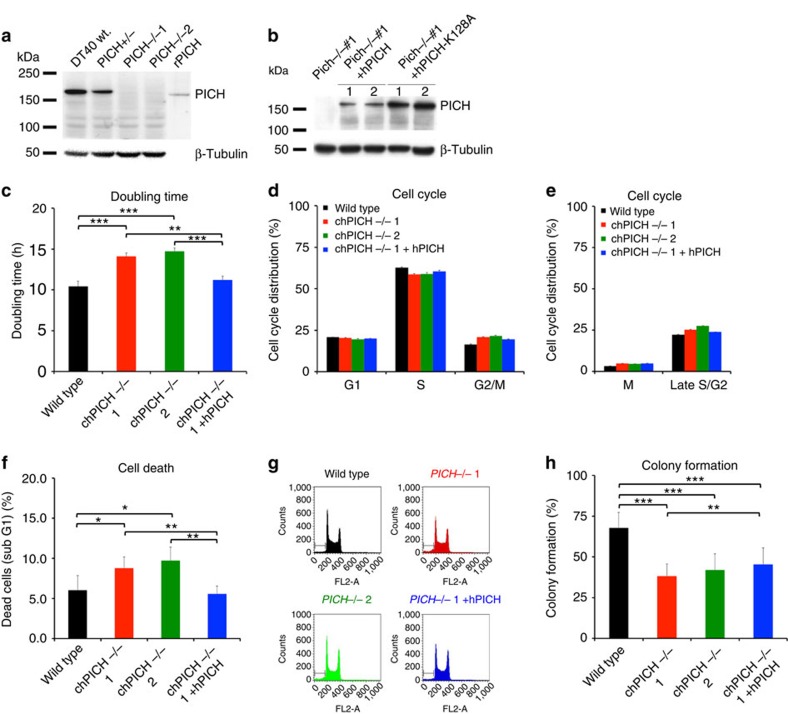
Characterization of PICH knockout and rescue cells. (**a**) Western blot of whole-cell extracts from wild type, *PICH*^+/−^ and two *PICH*^*−/−*^ clones probed with a PICH-specific antibody. Purified recombinant human PICH (rPICH) is shown as control. β-Tubulin was used as a loading control. (**b**) Western blot of whole-cell extracts from *PICH*^*−/−*^ cells stably transfected with human PICH fused to mCherry (hPICH-mCherry), or a catalytically dead human PICH (hPICH-K128A-Cherry). β-Tubulin was used as a loading control. (**c**) Doubling time of the indicated cell lines during exponential growth. (**d**) Cell cycle distribution of asynchronously growing cells of the indicated genotypes analysed by PI-FACS/DNA content. (**e**) FACS distribution of asynchronously growing cells in mitosis and late S/G2, determined by immunofluorescent staining of cells with the MPM-2 antibody (a mitotic marker) combined with PI staining of DNA. (**f**) Spontaneous cell death of cells of the indicated genotypes was analysed by quantifying the PI FACS/DNA sub-G1 content. (**g**) Examples of scored FACS profiles in **d** and **f**. (**h**) Colony survival of the indicated cell lines in methylcellulose media. (**c**–**f** and **h**) Each data point is an average of at least three independent experiments ±s.d. Significance levels were determined using the Student's *t*-test for parametric observations and are indicated as *P<0.05, **P<0.01 and ***P<0.001.

We identified the *Gallus gallus PICH* gene through database searches as an open reading frame located on chicken chromosome 4. The *PICH* gene encodes a protein of 1,280 amino acids with a calculated molecular mass of 144 kDa. Alignment of the predicted chicken and human PICH (hPICH) protein sequences revealed strong similarity (58.2% overall), including the conservation of the ATPase domain, the so-called PICH family domain^8^ and the two tetratricopeptide repeat motifs (Fig. 1a). We generated two independent *PICH*^*−/−*^ DT40 cell lines by targeted inactivation of both *PICH* alleles, as described in the Methods section and Fig. 1b. We verified that gene targeting was successful by a combination of Southern blotting, PCR analysis and western blotting using an anti-PICH antibody that recognizes both human and avian PICH (Figs 1c,e and 2a,b).

### Chromosomal instability of PICH^*−/−*^ cells

The *PICH*^*−/−*^ cell lines exhibited a mild, but consistent, proliferation defect with an average doubling time ∼25% longer than that of parental cells (Fig. 2c). This phenotype was associated with a modest extension of cell cycle transit time (Fig. 2d/e), combined with an approximately two-fold increase in the frequency of cell death (Fig. 2f/g). The colony-forming ability of the *PICH*^*−/−*^ cells was also reduced, as compared with that of the parental cells (Fig. 2h).

**Figure 3:**
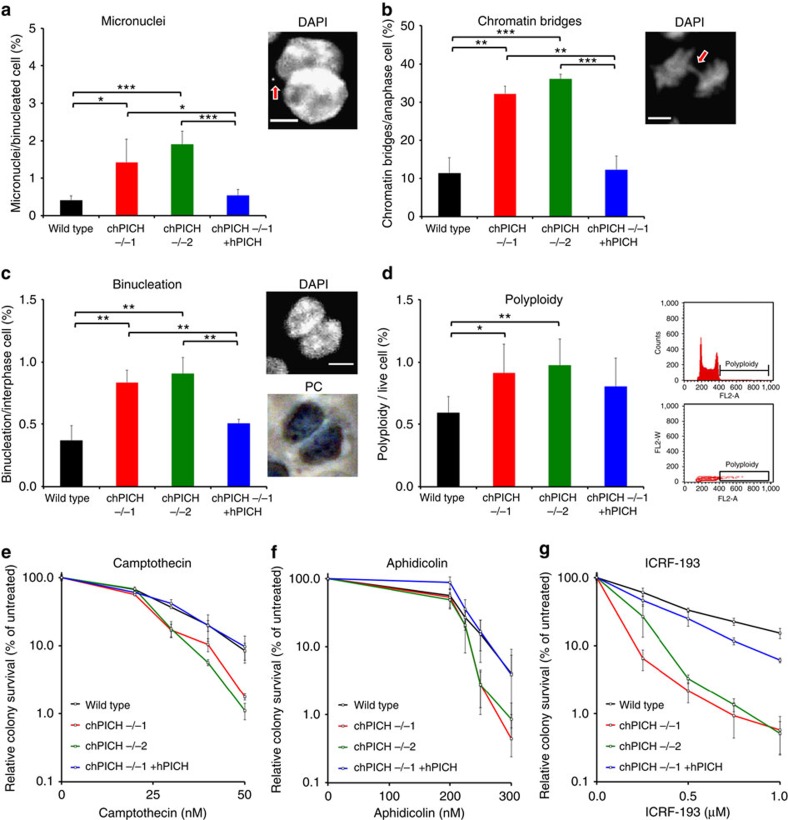
Chromosomal instability and drug sensitivity of *PICH*^*−/−*^ cells. (**a**) Frequency of micronucleus formation in binucleated cells arrested at cytokinesis with cytochalasin B and stained with DAPI. An example of a scored micronucleus is shown (red arrow). (**b**) Frequency of chromatin bridge formation in anaphase cells stained with DAPI. An example of a scored bridge is shown (red arrow). (**c**) Frequency of binucleation in asynchronously growing cells stained with DAPI and imaged by fluorescence and phase contrast microscopy. An example of a scored binucleated cell is shown. (**a**–**c**) Scale bars, 5 μm. (**d**) Frequency of polyploidy in cells stained with propidium iodide. Cells with a greater than G2/M DNA content were quantified using FACS. An example of a FACS profile is shown with the position of polyploid cells marked. Examples of profiles for all cell lines are presented in Supplementary Fig. 1a. The setting used for gating away dead cells and aggregates is shown in Supplementary Fig. 1b. In all cases, each data point is an average of at least three independent experiments ±s.d. Significance levels were determined using the Student's *t*-test for parametric observations, and are indicated as *P<0.05, **P<0.01 and ***P<0.001. (**e**–**g**) Clonogenic survival assays. Cells of the indicated genotypes were exposed continuously to camptothecin (**e**), aphidicolin (**f**) or ICRF-193 (**g**). Each data point is the average of at least three independent experiments performed in triplicate ±s.d.

Because PICH knockdown in human cells increases the occurrence of chromosomal abnormalities, such as chromatin bridges and micronuclei^13^,^14^,^16^,^17^, we analysed whether PICH-deficient DT40 cells displayed an altered frequency of spontaneous chromosomal abnormalities. For this, we analysed four widely studied markers of chromosomal instability (Fig. 3a–d and Supplementary Fig. 1a/b). We observed that the *PICH*^*−/−*^ cells had a significantly elevated frequency of (i) micronucleus formation, (ii) chromatin bridging (bridges that are both histone- and DAPI-positive in anaphase), (iii) binucleation (cells with two decondensed daughter nuclei in the same plasma membrane^21^) and (iv) polyploidy. In all cases, the *PICH*^*−/−*^ phenotypes were completely or partially corrected by ectopic expression of hPICH.

Treatment of human cells with ICRF-193, a catalytic inhibitor of Topo II, increases the number of PICH-positive UFBs^10^,^11^. However, the survival of PICH-deficient cells in response to cytotoxic agents has not been directly analysed previously. Hence, we compared the sensitivity of parental versus *PICH*^*−/−*^ chicken DT40 cells to a variety of cytotoxic agents using clonogenic survival assays. The *PICH*^*−/−*^ cells showed the same level of sensitivity as parental cells to the DNA-damaging agents cisplatin, methyl methane-sulfonate and mitomycin C (Supplementary Fig. 1c,e). However, the *PICH*^*−/−*^ cells showed mild sensitivity to camptothecin and aphidicolin (<1.5-fold in each case, based on the dose required to kill 90% of cells; Fig. 3e/f), and a more striking sensitivity to ICRF-193 (4.7-fold; Fig. 3g and Supplementary Fig. 1f). The increased sensitivity of *PICH*^*−/−*^ cells to these agents was confirmed to be due to the absence of PICH, as these phenotypes were corrected by ectopic expression of hPICH (Fig. 3e/g). Although ICRF-193 predominantly exerts its cytotoxic effects in mitosis, the sensitivity of *PICH*^*−/−*^ cells to this agent does not appear to reflect a general mitotic defect. In support of this proposal, the *PICH*^*−/−*^ cells did not show hypersensitivity to microtubule-disrupting agents such as nocodazole and paclitaxel (Supplementary Fig. 1g/h), indicating that the spindle assembly checkpoint is not compromized by loss of PICH.

### ICRF-193 causes mitotic defects in *PICH* ^*−/−*^ cells

**Figure 4:**
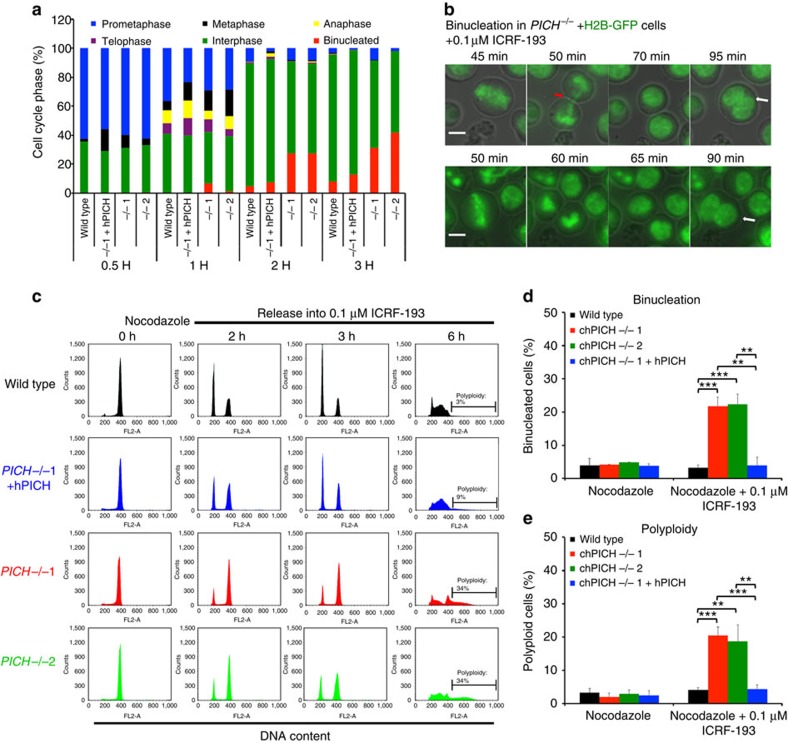
Mild Topo II inhibition causes *PICH*^*−/−*^ cells to abort mitosis. (**a**–**e**) Cells of the indicated genotypes were released from a nocodazole-induced prometaphase arrest into the latter stages of mitosis in medium containing 0.1 μM ICRF-193. (**a**) Quantification of mitotic progression into interphase or binucleation determined by immunofluorescent staining of β-tubulin combined with DAPI staining of DNA. (**b**) *PICH*^*−/−*^ cells stably expressing H2B-GFP were followed by time-lapse live cell microscopy. Representative images of cells becoming binucleated are shown. Red arrows denote chromatin bridges in mitosis and white arrows binucleated cells. Scale bars, 5 μm. The corresponding time-lapse movie is supplied as Supplementary Movie 1. (**c**) PI-FACS profiles of the indicated cell lines at different time points after release from a nocodazole arrest into medium containing ICRF-193. (**d**) Quantification of binucleation by fluorescence and phase contrast microscopy of the indicated cell lines stained with DAPI 6 h after release from nocodazole arrest. (**e**) Quantification of polyploidy by PI-FACS in the indicated cell lines 6 h after release from a nocodazole arrest. Polyploidy was quantified as shown in Supplementary Fig. 1a,b. (**d**–**e**) Each data point is an average of at least three independent experiments ±s.d. Significance levels were determined using the Student's *t*-test for parametric observations, and are indicated as *P<0.05, **P<0.01 and ***P<0.001.

To further examine the causes of ICRF-193-induced lethality in *PICH*^*−/−*^ cells, we examined the consequences of treating *PICH*^*−/−*^ cells with ICRF-193 specifically during mitosis. To achieve this, we synchronized cells with nocodazole in prometaphase and then released them from this arrest into medium containing a low dose (0.1 μM) of ICRF-193. We then followed the progression of the cells through the remainder of mitosis and into the next interphase over a period of 6 h. DAPI staining of mitotic nuclei and immunofluorescent staining for β-tubulin revealed that both the *PICH*^*−/−*^ and the hPICH-complemented cells progressed normally into metaphase, but then the *PICH*^*−/−*^ cells exhibited an abnormal anaphase DNA morphology suggestive of defective sister chromatid segregation (Supplementary Fig. 2a). For example, after 2 h, when >90% of the hPICH-complemented cells had progressed into G1-phase of the next cell cycle, ∼25% of the *PICH*^*−/−*^ cells exhibited a binucleated morphology (Supplementary Fig. 2a and Fig. 4a). Live-cell imaging of *PICH*^*−/−*^ cells stably expressing green fluorescent protein (GFP)-tagged histone H2B revealed that some, but not all, of the *PICH*^*−/−*^ anaphase cells contained a bulky chromatin bridge before becoming binucleated (Fig. 4b and Supplementary Movie 1). The remaining cells that were destined to become binucleated appeared to undergo a normal anaphase. However, as discussed further below, these cells harbour unresolved UFBs that are undetectable under these experimental conditions.

Flow cytometry traces for the ICRF-193-treated *PICH*^*−/−*^ cells (Fig. 4c and Supplementary Fig. 2b) revealed a pronounced peak of cells with a 4N DNA content after 2–3 h of treatment, suggestive of an apparent failure of the *PICH*^*−/−*^ cells to properly exit mitosis in the presence of ICRF-193. However, microscopic analysis revealed that this 4N population largely consisted of cells that had progressed into a subsequent G1 phase in a binucleated state (Fig. 4a). ICRF-193-treated *PICH*^*−/−*^ cells also exhibited a striking increase in the frequency of polyploid cells after 6 h of treatment (34%, as compared with 3% for parental cells and 9% for hPICH-complemented cells; Fig. 4c) suggesting that many of the binucleated cells entered the next S-phase and underwent re-replication. Consistent with this interpretation, when the ICRF-193-treated *PICH*^*−/−*^ cells were followed through a second S phase, a G2/M peak indicative of a tetraploid population (with an 8n DNA content) was observed (Supplementary Fig. 2c). Quantification of the frequency of ICRF-193-induced binucleation and polyploidization revealed that the *PICH*^*−/−*^ cells exhibited an approximately five-fold elevated frequency of both binucleated and polyploid progeny (Fig. 4d/e). These data define a hypersensitivity of *PICH*^*−/−*^ cells to ICRF-193-induced mitotic defects. The concentration of ICRF-193 had to be increased ten-fold (to 1 μM; Supplementary Fig. 2d) in order to generate levels of polyploidization above background in parental cells. Moreover, *PICH*^*−/−*^ cells released from nocodazole arrest into drug-free medium did not show evidence of a significant level of binucleation/polyploidization (Supplementary Fig. 2e and Fig. 4d,e), indicating that ICRF-193 treatment strongly induces these phenotypes. Taken together, we propose that PICH-deficient cells are highly sensitive to inhibition of Topo II in mitosis, and that Topo II function is crucial for the prevention of binucleation and polyploidization in DT40 cells. Exposure of *PICH*-deficient cells to aphidicolin or camptothecin in interphase to induce replication stress and DNA damage did not increase the frequency of polyploidization above that of untreated controls (Supplementary Fig. 3a,b), suggesting that the induction of polyploidization in ICRF-193-treated *PICH*^*−/−*^ cells specifically arises as a consequence of defective DNA decatenation in mitosis.

### The PICH ATPase is required for efficient UFB resolution

**Figure 5:**
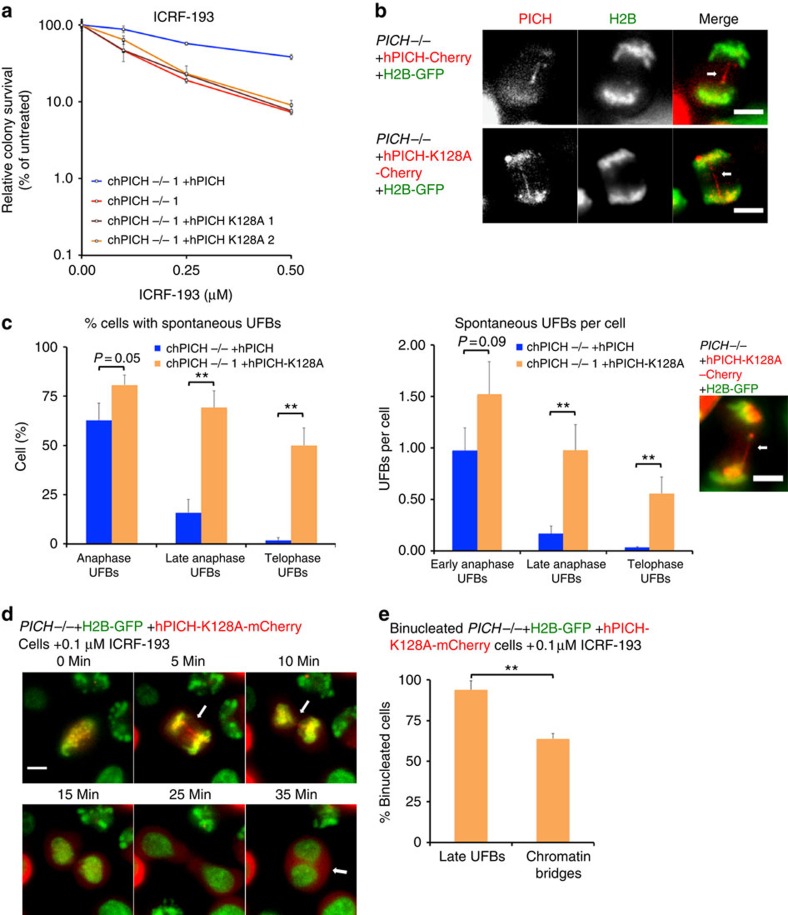
*PICH*^*−/−*^ cells expressing ATPase-dead PICH have defective UFB resolution. (**a**) Clonogenic survival assay. Cells of the indicated genotypes were treated with ICRF-193. Each data point is the average of at least three independent experiments performed in triplicate ±s.d. (**b**) Cells were arrested in prometaphase with nocodazole and released. Arrows denote UFBs. Scale bars, 5 μm UFBs were visualized by Z-stacked, time-lapse live cell microscopy of *PICH*^*−/−*^ +H2B-GFP (green) cells stably expressing either hPICH-mCherry (red) or hPICH-K128A-mCherry (red). (**c**) Quantification of the percentage of cells harbouring UFBs (left) and the number of UFBs per cell (right) in early anaphase, late anaphase and telophase cells. An example of a telophase UFB is shown (far right). Each data point is an average of at least three independent experiments ±s.d. Significance levels were determined using the Student's *t*-test for parametric observations, and are indicated as *P<0.05, **P<0.01 and ***P<0.001. (**d**) *PICH*^*−/−*^ cells stably expressing H2B-GFP (green) and hPICH-K128-mCherry (red) were followed by time-lapse live cell microscopy after release from nocodazole arrest into medium containing 0.1 μM ICRF-193. White arrows denote UFBs during mitosis or binucleation. Scale bar, 5 μm. Representative images of a cell becoming binucleated are shown. The corresponding time-lapse movie is supplied as Supplementary Movie 2. (**e**) Quantification of chromatin bridges and UFBs in the mitosis of cells that become binucleated. Each data point is an average of at least three independent experiments ±s.d. Significance levels were determined using the Student's *t*-test for parametric observations, and are indicated as *P<0.05, **P<0.01 and ***P<0.001.

Previous studies have indicated that ectopic expression of ATPase-dead PICH is unable to prevent the excessive chromatin bridge formation associated with depletion of PICH from human cells^17^. To investigate further the functional role of the PICH ATPase activity, we compared the phenotypes of *PICH*^*−/−*^ cells stably expressing either mCherry-tagged hPICH or hPICH-K128A, which contains a mutation in the Walker A box that is known to abolish ATP-hydrolysis^12^. Consistent with an important role for PICH ATPase activity in mitosis, we observed that the hypersensitivity of the *PICH*^*−/−*^ cells to ICRF-193 was rescued by expression of hPICH, but not hPICH-K128A (Fig. 5a). We therefore investigated the localization of the hPICH and hPICH-K128A proteins in mitosis using live-cell imaging. We observed that both hPICH and hPICH-K128A localized to UFBs in DT40 cells, indicating that PICH ATPase activity is not required to establish the presence of PICH on UFBs (Fig. 5b). These data also indicate that UFBs cannot be generated from chromatin bridges via the removal of histones by the ATP-driven translocase activity of PICH.

Next, we quantified the frequency of spontaneous UFBs in the *PICH*^*−/−*^ cells expressing either hPICH or ATPase-dead hPICH. In early anaphase, the percentage of cells displaying UFBs was only marginally higher in those *PICH*^*−/−*^ cells expressing hPICH-K128A (79%), as compared with those expressing hPICH (63%). However, a more pronounced difference was apparent in late anaphase and telophase cells, as cells expressing ATPase-dead hPICH exhibited a strikingly higher overall frequency of UFBs (Fig. 5c). Based on these data, we propose that the ATPase activity of PICH is not required to suppress UFB formation, but it is required for the efficient resolution of UFBs during anaphase and telophase. This observation clarifies some of the uncertainty in previous reports regarding the roles of the PICH ATPase activity^11^. We confirmed that the persistent UFBs present in cells expressing hPICH-K128A protein displayed foci for the kinetochore-associated CENPA protein at their termini (Supplementary Fig. 3c). Interestingly, the persistence of UFBs in cells expressing ATPase-dead hPICH did not affect the overall timing of progression from prometaphase to telophase, as cells expressing either hPICH or hPICH-K128A showed similar rates of mitotic progression (Supplementary Fig. 3d). Expression of hPICH-K128A in the *PICH*^*−/−*^ cells also did not rescue the extended doubling time (Supplementary Fig. 3e), increased chromatin bridges (Supplementary Fig. 3f) or binucleation phenotypes of *PICH*^*−/−*^ cells (Supplementary Fig. 3g), suggesting that the ATPase activity of PICH is important for promoting chromatid disjunction in mitosis.

### UFBs can cause cytokinesis failure and polyploidization

Next, we sought to investigate the effects of unresolved UFBs on the latter stages of mitosis. Using live imaging of *PICH*^*−/−*^ cells expressing hPICH-K128A-mCherry, we investigated whether unresolved UFBs in telophase might lead to binucleation and/or cytokinesis failure. This was undertaken because, as discussed above, only a proportion of the *PICH*^*−/−*^ cells that became binucleated contained a bulky chromatin bridge in anaphase (Fig. 4). To analyse this, we followed the progression of cells from the point of release from a nocodazole arrest into medium containing 0.1 μM ICRF-193 (Fig. 5d and Supplementary Movie 2). We observed that ∼60% of the anaphases that subsequently generated binucleated cells contained a chromatin bridge (Fig. 5e). In contrast, the vast majority (>90%) of these cells displayed at least one UFB that persisted into telophase, before cytokinesis was aborted and the cells became binucleated. This observation, in conjunction with the data shown in Fig. 4, argue that unresolved UFBs can also trigger cytokinesis failure, binucleation and polyploidy. This is the first demonstration of a pathological consequence of a failure to specifically resolve UFBs in late mitosis, as all previous studies have been unable to distinguish between the effects of an elevated number of UFBs versus a delay in their resolution.

### PICH bodies form at decondensed rDNA in mitosis

**Figure 6:**
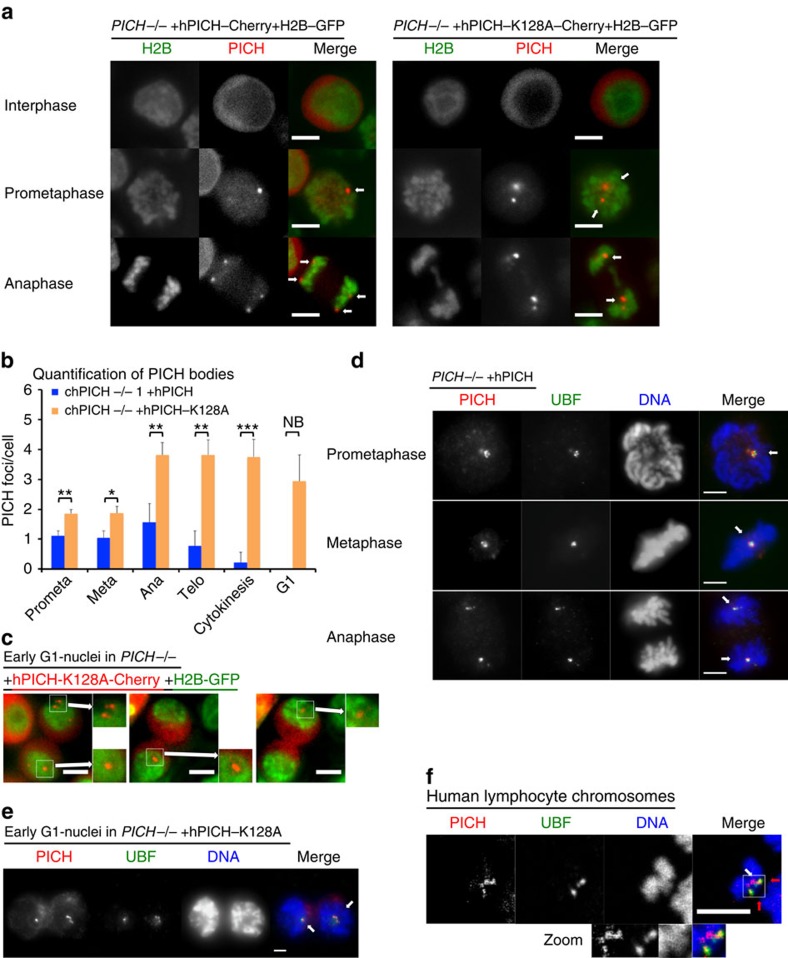
PICH forms ‘PICH Bodies' on decondensed rDNA in mitosis. (**a**–**e**) Cells were arrested in prometaphase by nocodazole treatment and then released. Scale bars, 5 μm. (**a**) Representative live cell images of *PICH*^*−/−*^ +H2B-GFP (green), +hPICH-mCherry (red) or hPICH-K128A-mCherry (red) cells in interphase and mitosis. White arrows denote PICH bodies. (**b**) Quantification of PICH bodies in mitosis of *PICH*^*−/−*^ cells expressing hPICH or hPICH-K128A. Each data point is an average of at least three independent experiments ±s.d. Significance levels were determined using the Student's *t*-test for parametric observations, and are indicated as *P<0.05, **P<0.01 and ***P<0.001. NB: Significance could not be determined in the *PICH*^*−/−*^ +hPICH cell line due to a lack of PICH foci in G1. (**c**) Representative live cell images of early G1 *PICH*^*−/−*^ +H2B-GFP (green) +hPICH-K128A-mCherry (red) cells. The zoomed images denoted by white boxes show nucleoli, containing PICH bodies. (**d**–**f**) PICH (red) and UBF (green) were immunofluorescently stained and DNA (blue) was stained with DAPI. (**d**) Representative images of *PICH*^*−/−*^ +hPICH cells in mitosis. (**e**) Representative microscopy images of early G1 *PICH*^*−/−*^ +hPICH-K128A cells. White arrows denote UBF-positive nucleoli containing PICH foci. (**f**) Single optical slices from confocal imaging of chromosome spreads from human lymphocytes treated with colcemid for 4 h. Red arrows denote UBF-PICH foci, and white arrows denote centromeric PICH foci. Zoomed images of the foci are shown in white boxes. The whole chromosome spread is presented in Supplementary Fig. 4d.

**Figure 7:**
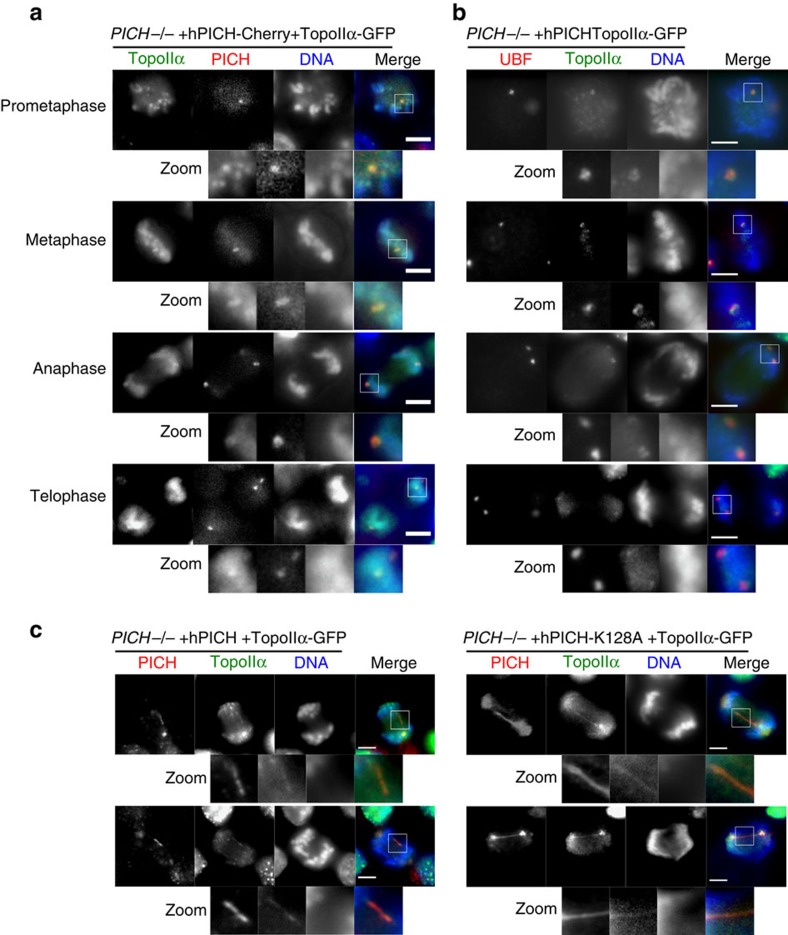
Topo IIα localizes with PICH in PICH bodies and on UFBs. (**a**–**c**) Cells were arrested in prometaphase using nocodazole, released and imaged by time-lapse or immunofluorescent microscopy. Scale bars, 5 μm. (**a**) Representative images of PICH bodies in *PICH*^*−/−*^ +Topo IIα-GFP (green), +hPICH-mCherry (red) cells in mitosis, stained with Hoechst 33342 (blue). (**a**–**b**) The zoomed images denoted by white boxes highlight PICH bodies. (**b**) Representative microscopy images of *PICH*^*−/−*^ +Topo IIα-GFP (green) +hPICH cells. UBF (red) was immunofluorescently stained and DNA (blue) was stained with DAPI. Arrows and zoomed images highlight UBF-Topo IIα foci. (**c**) Representative microscopy images of UFBs in *PICH*^*−/−*^ +Topo IIα-GFP (green) +hPICH- or +hPICH-K128A cells. PICH (red) was immunofluorescently stained and DNA (blue) was stained with DAPI. The zoomed images denoted by white boxes highlight UFBs.

Consistent with previous findings in human cells^8^,^9^, PICH and PICH-K128A were retained in the cytoplasm during interphase in chicken cells and only gained access to chromatin in mitosis (Fig. 6a). During immunofluorescence and live-cell imaging analysis of these mitotic cells, we observed that, in addition to its expected localization to UFBs, PICH was also detectable on another prominent sub-nuclear structure (Fig. 6a and Supplementary Figs 3d and 4a,b). This pattern was detected consistently in wild-type cells (Supplementary Fig. 4a), as well as in cells expressing hPICH-mCherry protein (Fig. 6a). This suggested that these large foci, which we have termed ‘PICH bodies', comprise *bona fide* physiological structures. The PICH bodies were always detected within non-condensed regions of DNA, and did not co-localize with H2B-GFP, DAPI, Hoechst 33342 or CENPA staining (Figs 6a and 7a and Supplementary Fig. 4a,b). Therefore, PICH bodies do not appear to define any form of condensed DNA, despite being associated with the DNA masses in mitosis. Time-lapse live-cell microscopy of *PICH*^*−/−*^ cells expressing H2B-GFP together with either hPICH-mCherry or hPICH-K128A-mCherry revealed that hPICH bodies gradually disappear during progression through mitosis (Fig. 6b). The ATPase-dead PICH similarly formed PICH bodies, but in this case they persisted for a longer period, often until the early G1 phase of the next cell cycle (Fig. 6b/c). Moreover, the frequency of PICH bodies was increased in cells expressing ATPase-dead PICH (Fig. 6b). We also observed that the number of PICH bodies increased during the period from metaphase through anaphase, as expected of a structure associated with the nuclear genome, and then segregated evenly in anaphase to the two daughter masses (Fig. 6b and Supplementary Fig. 4c).

Because the PICH bodies containing hPICH-K128A often persisted until the cells entered the next G1 phase (Supplementary Movie 3), and localized to regions in the nucleus that were noticeably less histone H2B-dense (Fig. 6c), it seemed likely that these hPICH-K128A bodies would define the location of newly formed nucleoli^22^. To test this hypothesis, we analysed whether PICH co-localizes with the rDNA transcription factor, UBF, which has been demonstrated previously to localize to the rDNA promoters in mitosis and interphase^10^,^23^. We observed that PICH bodies always co-localized with UBF in mitotic cells (Fig. 6d). Moreover, the persistent hPICH-K128A bodies evident in early G1 cells co-localize with UBF, indicating that they were coincident with nucleoli and rDNA (Fig. 6e). Next, we investigated whether PICH bodies are also observed in human cells. We observed that PICH and UBF proteins co-localized on human metaphase chromosomes spreads (Fig. 6f). In this case, we could also distinguish the rDNA-associated UBF/PICH foci from the centromeric PICH foci detectable on the same chromosomes, eliminating the possibility that the PICH-UBF co-localization could be due simply to the close proximity of centromeres and rDNA loci on acrocentric human chromosomes. Moreover, we observed that UBF foci did not generally co-localize with DAPI-stained DNA, unlike centromeric PICH foci (Fig. 6d/f, Supplementary Fig. 4d^8^,^10^). We note, however, that PICH did not co-localize with all UBF foci, suggesting that PICH may associate with rDNA loci in a transient manner. This proposal is consistent with the finding that PICH bodies observed in DT40 cells expressing hPICH gradually disappeared during or soon after anaphase (Fig. 6b).

### PICH and Topo IIα co-localize at PICH bodies and UFBs

Given that the phenotype of PICH-deficient cells resembles that of cells defective in Topo II function, we examined if PICH might modulate Topo II-mediated sister chromatid decatenation. To achieve this, we first integrated sequences encoding a GFP tag at the endogenous *TOP2A* locus in the different *PICH*^*−/−*^ cell lines. The expression of GFP-tagged Topo IIα was confirmed by immunoblotting for GFP and Topo IIα (Supplementary Fig. 5a). As expected, Topo IIα-GFP was distributed widely on chromatin, but was enriched in PICH bodies (marking rDNA; as defined by UBF staining) from prometaphase to telophase (Fig. 7a,b and Supplementary Fig. 5b). Topo IIα also co-localized with PICH on UFBs, although its distribution along the bridges was often less homogeneous (Fig. 7c). To extend this observation, we performed time-lapse microscopy in the absence of DNA staining. We observed that Topo IIα was detectable on PICH-positive DNA bridges, and that both proteins disappeared simultaneously from these bridges during anaphase (Supplementary Fig. 5c). A similar association on DNA bridges was also observed in cells expressing ATPase-dead PICH (Supplementary Fig. 5c), although in this case dissociation of Topo IIα was often seen to precede that of PICH.

### Abnormal chromosome structure in PICH-deficient human cells

**Figure 8:**
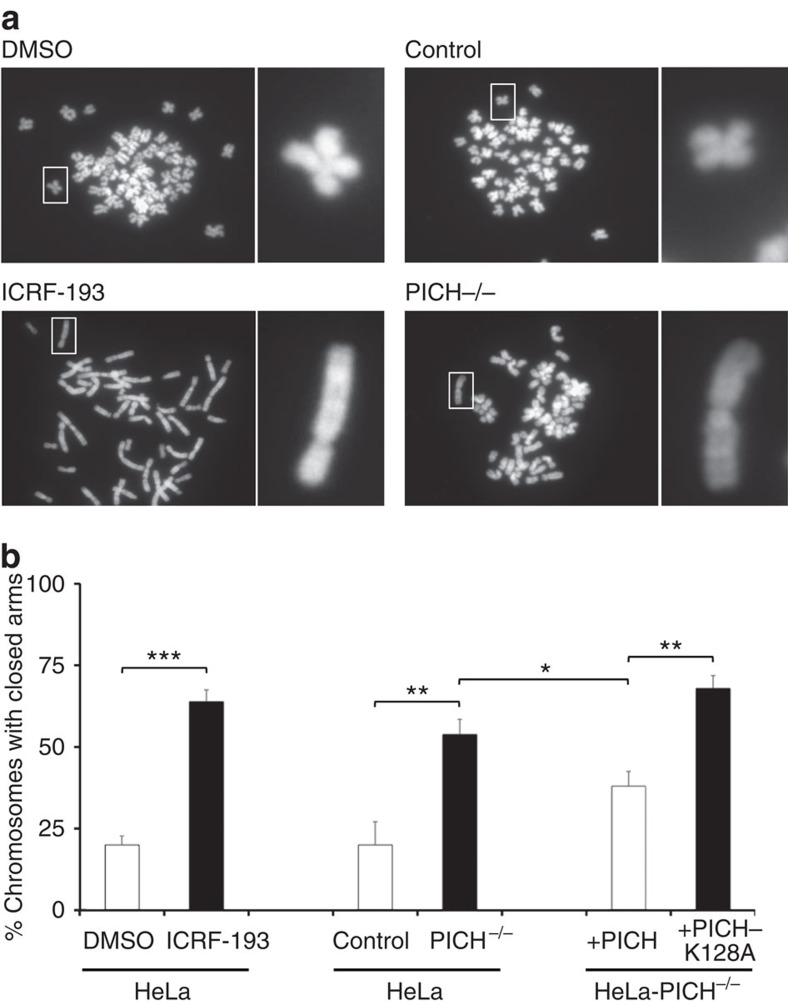
Human *PICH*^*−/−*^ cells have condensation and cohesion defects. (**a**–**b**) Increased sister chromatid cohesion and reduced condensation detected in control cells treated with 0.1 μM ICRF-193 and in *PICH*^*−/−*^ human cells. Cells were synchronized by thymidine for 24 h and released into nocodazole for 14 h, followed by spreading onto glass slides and counterstaining with DAPI. For rescue experiments, *PICH*^*−/−*^ cells were transfected with wild-type or K128A mutant *PICH* for 18 h before cells were synchronized using thymidine. Percentage of cells with closed and stretched chromosome arms is shown. Each data point is an average of at least three independent experiments ±s.d. Significance levels were determined using the Student's *t*-test for parametric observations, and are indicated as *P<0.05, **P<0.01 and ***P<0.001. (**a**) White boxes denote zoomed images of chromosomes in open or closed-arm conformation (shown on the right).

To strengthen and extend the proposal that PICH co-operates with Topo IIα in effecting sister chromatid decatenation, we generated a PICH-deficient human cell line (derived from HeLa cells) using CRISPR-Cas9-mediated gene disruption (Methods). We observed that the PICH-deficient human cells showed a similar spectrum of chromosome instability in mitosis as is seen in *PICH*^*−/−*^ DT40 cells, including micro- and bi-nucleation (Supplementary Fig. 6). However, the primary reason for generating a *PICH*^*−/−*^ human cell was to permit a more robust analysis of metaphase chromosome structure, which is technically easier with human than with chicken chromosomes. An established consequence of Topo IIα inhibition by ICRF-193 is the failure of the chromosomes in metaphase to adopt the normal hyper-condensed, X-shaped morphology. Instead, metaphases from ICRF-193-treated cells show an excess of under-condensed chromosomes that do not appear to have undergone complete arm decatenation (giving a ‘closed-arm' appearance; Fig. 8). We observed that metaphases from the PICH knockout human cells contained a significantly higher frequency of chromosomes with an undercondensed, closed-arm appearance than did the control metaphases (Fig. 8). This abnormality was partially suppressed by ectopic expression of the wild-type PICH protein, but not the PICH-K128A protein (Fig. 8). We conclude, therefore, that PICH-deficient human cells generate metaphase chromosomes with an appearance similar to that of ICRF-193-treated normal cells, consistent with a defect in sister chromatid condensation and decatenation before anaphase.

### PICH stimulates Topo IIα catalytic activity *in vitro*

**Figure 9:**
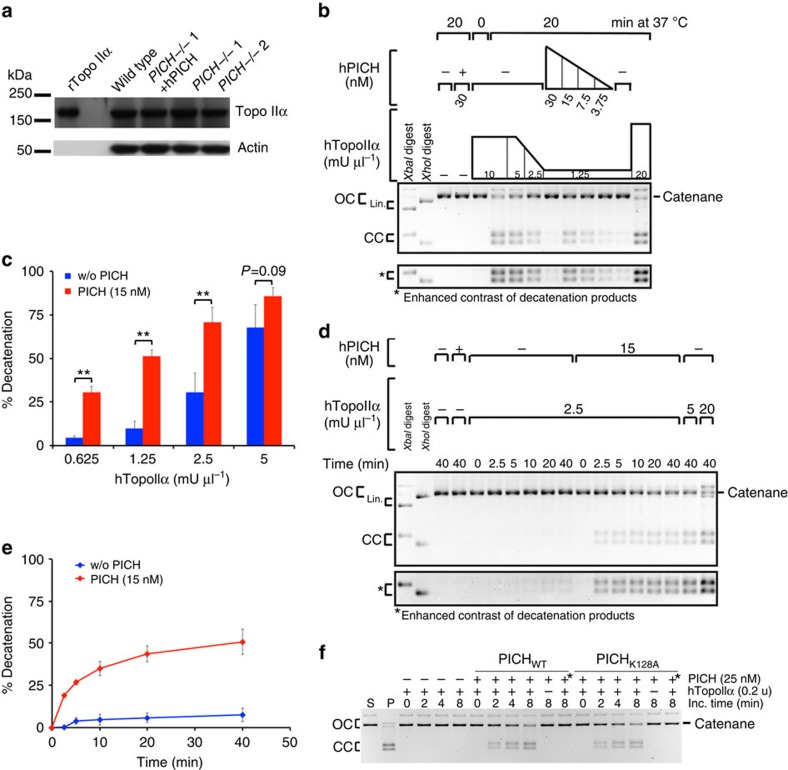
PICH stimulates Topo IIα decatenation activity on a single catenane substrate. (**a**) Western blotting analysis of purified recombinant Topo IIα (left lane) and mitotic nuclear extracts from cells of the indicated genotypes. Actin was used as a loading control. (**b**,**d**,**f**) Representative gel images of decatenation assays combining human Topo IIα and purified recombinant hPICH with a single catenane substrate. DNA products are denoted as follows: lin, linearized DNA; OC, open circular (nicked substrates); CC, closed circular decatenated DNA. (**b**) Decatenation assay analysing PICH concentration range with a fixed Topo IIα concentration. (**c**) Quantification of decatenation products with the indicated Topo IIα concentrations in the absence (blue) or the presence (red) of 15 nM PICH. Each data point is an average of at least three independent experiments ±s.d. Significance levels were determined using the Student's *t*-test for paired parametric observations, and are indicated as **P<0.01. (**d**) A representative time course for decatenation assays carried over 40 min with the indicated concentrations of Topo IIα (with or without addition of PICH). Samples were taken at the indicated time points. (**e**) Quantification of decatenation products from the time course assays with 2.5 mU μl^−1^ Topo IIα in the absence or presence of PICH. Each data point is an average of three independent experiments ±s.d. (**f**) A representative time course assay of single catenane substrate decatenation by Topo IIα with or without PICH (PICH_WT_) or ATPase-dead PICH (PICH_K128A_). Incubations were terminated at the indicated time points. S, substrate; P, product (obtained by incubating the substrate under the same conditions but with 2u of Topo IIα); +*: Indicates the lane in which PICH has been heat inactivated at 95 °C for 5 min before the reaction.

Thus far, our data indicate that PICH deficiency partially ‘phenocopies' inhibition of Topo II decatenation activity, suggesting that PICH might regulate Topo II activity *in vivo*. A trivial explanation for the effects reported here could be that the *PICH*^*−/−*^ cells express lower levels of Topo IIα. However, western blotting of mitotic cell extracts revealed that all of the cell lines examined in this study expressed similar levels of Topo IIα (Fig. 9a and Supplementary Fig 7a). We therefore investigated whether PICH might function as a co-factor for Topo IIα. To do this, we analysed whether recombinant PICH protein could influence the decatenation activity of Topo IIα *in vitro*. Topo IIα activity is generally assayed using kinetoplast DNA as a substrate, but this assay is only semi-quantitative because the kinetoplast DNA comprises multiple interlocked monomer rings that are retained in the wells of the gels, and cannot be released as free monomers until several independent decatenation steps have been undertaken. To develop a more quantitative assay for Topo II, we used the Tn3 site-specific recombinase to generate a single catenane substrate that comprises two interlocked plasmids that can be distinguished from each other by their size (Supplementary Fig. 7b,c and Methods). Using this substrate, we observed that PICH was able to strongly stimulate Topo IIα-mediated decatenation in both a Topo IIα concentration gradient (Fig. 9b/c) and a time course (Fig. 9d/e). Surprisingly, ATPase-dead PICH also stimulated Topo IIα activity to the same extent as PICH (Fig. 9f). This was seen with multiple preparations of PICH/PICH-K128A proteins that were purified with or without a GFP tag. The stimulation by PICH was particularly striking in assays where low concentration of Topo IIα was utilized, but was significant at all Topo IIα concentrations analysed (Fig. 9c). Importantly, heat inactivation of any of the PICH preparations resulted in a failure to stimulate Topo IIα. To analyse whether this stimulation was specific for the decatenation activity of Topo IIα, or rather was a reflection of a general stimulation of catalytic activity, we assessed whether PICH could stimulate the relaxation of negatively supercoiled plasmid DNA by Topo IIα. We observed that both PICH and PICH-K128A were also able to stimulate the plasmid relaxation activity of Topo IIα (Supplementary Fig. 7d). When this assay was performed in the presence of an excess of either Topo IIα or Wheat Germ Topoisomerase I, the degree of supercoiling reached at the end of the reaction was not affected by the presence of PICH, indicating that PICH binding to DNA *per se* does not influence substrate topology (Supplementary Fig. 7e). We conclude, therefore, that PICH can directly stimulate Topo IIα catalytic activity.

## Discussion

The process of mitosis is probably the most vulnerable stage of the cell division cycle because chromosomes can be damaged, lost or unevenly segregated between the two daughter cells. Through analysis of chicken cells lacking the PICH protein, we have provided evidence that PICH and Topo IIα functionally cooperate in order to preserve chromosomal integrity during mitosis. Through the complementation of *PICH*^*−/−*^ DT40 cells with fluorescently tagged hPICH proteins, we were also able to visualize the behaviour of PICH and PICH-K128A during mitosis *in vivo*. As reported previously in human cells, we observed that PICH is excluded from the nucleus during interphase in DT40 cells and associates with centromeres in prometaphase, and DNA bridges connecting the separating sister chromatids in anaphase^8^,^9^. Interestingly, during our analyses, we also observed a novel type of sub-nuclear structure that we called PICH bodies. In cells expressing ATPase-dead PICH, these PICH bodies persisted through mitosis, and appeared inside newly formed nucleoli in G1 cells. Although these large PICH bodies are not so evidently detectable in human cells, we demonstrated that PICH co-localizes with a subset of UBF foci that mark rDNA loci in mitosis^10^,^23^ in human lymphocytes. We propose, therefore, that PICH has a hitherto unreported association with the rDNA loci in both chicken and human cells, suggesting an evolutionarily conserved role for PICH in the maintenance of rDNA stability. The difference in specific PICH-rDNA localization patterns between species can most likely be reconciled by the fact that the multiple rDNA units are dispersed on the short arm of the five acrocentric chromosomes in humans^24^, whereas the rDNA is present in a single gene cluster on chromosome 16 in chickens^25^. This also explains the larger size of the PICH bodies in chicken cells. The rDNA localization of PICH in human cells may also have been overlooked previously due to the close proximity of the rDNA and centromeres on the acrocentric chromosomes. Importantly, however, we could distinguish between PICH-UBF rDNA foci and centromeric PICH foci on the same chromosome, verifying that these are two distinct classes of PICH-associated loci. Furthermore, unlike centromeres, the PICH bodies in DT40 cells, and the PICH-UBF foci in human lymphocytes, are DAPI negative. Our observations are consistent therefore with previous observations that rDNA units are organized into decondensed nucleolar organizer regions during mitosis^26^.

Consistent with a functional interaction between PICH and Topo II, we also observed that Topo IIα co-localizes with PICH on UFBs and in PICH bodies. Based on the co-localization to PICH bodies, it is possible that PICH assists Topo IIα in the resolution of rDNA catenanes during anaphase. Indeed, in budding yeast, the rDNA array is decondensed and remains catenated until activation of the Cdc14 phosphatase by Separase at the metaphase-to-anaphase transition^27^. Top2 is also present on anaphase bridges in yeast^19^. Because UFBs have been proposed to contain catenated DNA, we consider it plausible that PICH stimulates Topo II to promote UFB resolution during mitosis.

We observed that PICH ATPase activity was required for normal UFB resolution and for the timely disappearance of PICH from PICH bodies. In *PICH*^*−/−*^ cells expressing ATPase-dead PICH, co-localization of the PICH-K128A and Topo II proteins to UFBs and PICH bodies was still detectable. However, unlike PICH-K128A, Topo II was not retained on unresolved UFBs that were detectable in telophase cells. Therefore, one possible role of PICH ATPase activity is to promote the retention of Topo II on those UFBs that persist into the later stages of mitosis. Interestingly, when PICH-K128A-expressing cells were treated in mitosis with ICRF-193, 35–40% of the cells abandoned cytokinesis to become binucleated. In >90% of these cells, a persistent UFB was present. These findings suggest that UFBs, like chromatin bridges and chromatin persisting in the midzone^28^, can trigger cleavage furrow regression, leading to binucleation and subsequent aneuploidy (reviewed in ref. 29). This observation argues that a defect in UFB resolution *per se* can directly cause chromosome segregation defects, and that these defects are unlikely to require any specific functions of histones on bridge DNA because UFBs are nucleosome free.

One unexpected finding, given that the ATPase activity of PICH is crucial for enzyme function *in vivo*, was the demonstration that ATPase-dead PICH effectively stimulated Topo II activity *in vitro*. There are several potential explanations for this. First, catenanes *in vivo* are likely to be far more complex topologically than the model single catenane substrate that we analysed *in vitro*. Second, the precursor DNA for UFBs is associated with nucleosomes *in vivo*, and it is possible that the ATPase function of PICH facilitates DNA decatenation prior to anaphase in the context of chromatin. Third, the substrate used *in vitro* can never fully recapitulate a catenated DNA structure that would be present in mitosis, in that it is not subjected to stretching forces. It will, therefore, be interesting to determine whether PICH ATPase activity is required for Topo II to efficiently decatenate stretched DNA molecules. In this regard, each step in the elaborate catalytic cycle of Topo II (DNA strand capture, cleavage, passage and re-ligation) could be influenced by the presence of tension on the catenanes. Finally, as discussed above, the PICH ATPase activity might be required to retain Topo II on those UFBs that persist into late anaphase or telophase.

**Figure 10:**
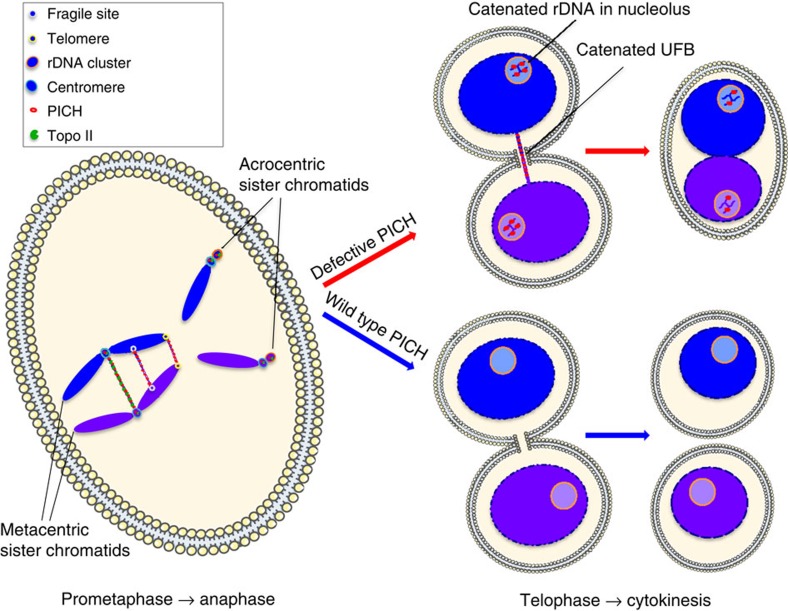
The roles of PICH in promoting faithful sister chromatid disjunction. In prometaphase, sister chromatids are held together by cohesin (not shown) only at the centromeric region. Centromeres are still linked by interchromatid catenanes because cohesin blocks the access of Topo IIα to these. PICH and Topo IIα co-localize at rDNA. At the onset of anaphase, centromeric cohesin is removed and sister chromatids begin to separate. Tension is applied to interchromatid DNA links and PICH binds strongly to these DNA bridges. PICH stimulates decatenation of catenated centromeric DNA bridges by Topo IIα. At the rDNA loci, anaphase onset permits condensation to occur in the rDNA, which allows decatenation by Topo IIα. In telophase and during cytokinesis, cells with defective PICH can have persistent DNA bridging at the midzone. This prevents cytokinesis, leading to binucleation. In cells expressing wild-type PICH, DNA bridge decatenation is completed through the action of Topo IIα to permit cell division. ATPase-dead/defective PICH localizes to nucleoli in telophase and early G1-phase cells. In these cells, ATPase-dead PICH fails to stimulate rDNA decatenation by Topo IIα efficiently. The rDNA in these cells have persistent catenations. In cells expressing wild-type PICH, the rDNA is sufficiently decatenated by the action of Topo IIα during anaphase and telophase.

In summary, we propose that PICH serves at least four important roles in promoting faithful chromosome segregation: (i) A sensor of stretched bridge DNA for rapid detection of UFBs as soon as anaphase is initiated. (ii) A stimulatory factor for the decatenation of UFBs by Topo IIα. (iii) A recruitment platform for other DNA repair factors, such as BLM and Topoisomerase III, which are perhaps able to process UFBs that are refractory to Topo IIα action. (iv) A stimulatory factor for the decatenation of rDNA by Topo IIα. A model depicting the cellular roles of PICH in eukaryotic cells is shown in Fig. 10. Future studies should aim to investigate the specific role of the PICH ATPase activity, and the consequences of PICH deficiency on rDNA stability and ribosomal biogenesis.

## Methods

### Cell culture and colony formation assays

Chicken DT-40 Cre1 B cell lymphoma cells were kindly supplied by Dr Minoru Takata (Kyoto, Japan) and were grown at 39.5 °C in RPMI-1640 GlutaMAX medium supplied with 10% fetal bovine serum, 2% chicken serum, penicillin/streptomycin (all from Gibco/Life sciences) and 50 μM 2-mercaptoethanol (Sigma). Human lymphocytes transformed with Epstein–Barr virus (GM06865, Cornell Institute) were grown in RPMI-1640 GlutaMAX medium supplemented with 15% fetal bovine serum and penicillin/streptomycin. Cytotoxic drugs used in the colony formation assays were from Sigma. Synchrony in prometaphase was achieved by treating cells with 0.05 μg ml^−1^ Nocodazole (Sigma) for 6.5 h. For live-cell imaging, cells were treated with 0.05 μg ml^−1^ Nocodazole for 5 h before release. For colony formation assays, single cells were seeded in DMEM F12 with L-glutamine (Gibco) supplemented with 10% FBS, 2% CS, 50 μM 2-mercaptoethanol, penicillin/streptomycin and 1.1% methylcellulose (Sigma), together with the indicated concentrations of the various drugs. Cells were grown for 8–10 days before colonies were counted.

### Construction of *PICH* targeting vectors

The sequence of the chicken *PICH* gene was obtained from the NCBI website. Genomic DNA from DT40 cells was isolated using extraction buffer (100 mM Tris-HCl, pH 7.5, 20 mM EDTA, 150 mM NaCl, 1% SDS and 200 μg ml^−1^ proteinase K) followed by Phenol–Chloroform extraction. The 5′ arm of the targeting vectors was amplified with SH-chPICH-10 and SH-chPICH-33 from DT-40 genomic DNA using a LA-PCR set (Takara). The 3′ arm was amplified with SH-chPICH-7 and SH-chPICH-8 using PrimeStarTM (Takara). PCR fragments were cloned into pBluescriptKS(+) and digested with *Not*I and *Eco*RI for the 5′ arm, and with *Bam*HI and *Sac*I for the 3′ arm. *Bsr-* or *Puro-*resistant cassettes were excised with *Eco*RI and *Bam*HI from cloning vectors. Homology arm fragments and either *Bsr* or *Puro* fragments were ligated with T4 DNA ligase (NEB) and cloned into the pBluescript vector.

### Establishment of *PICH* knockout cell lines

Thirty micrograms of the targeting vectors were linearized with *Ahd*I, precipitated with ethanol and then dissolved in 50 μl of PBS. The vectors were then electroporated into 1 × 10^6^ DT40Cre1 cells using a Bio-Rad electroporator set at 25 μF and 550 V. After overnight incubation in growth medium, cells were plated on 96-well plates and transfectants were selected using 25 μg ml^−1^ Blasticidin (first allele; Sigma) or 0.5 μg ml^−1^ Puromycin (second allele; Sigma) at 37 °C for 10–14 days. Selected clones were further incubated in six-well plates. Genomic DNA was extracted with DNA extraction buffer and phenol–chloroform extraction. DNAs were digested with *Bam*HI or *Spe*I at 37 °C overnight, precipitated with ethanol and then dissolved with 20 μl of TE. 5′ and 3′ flanking probes were used to detect correctly targeted clones. Knockout clones were also confirmed by PCR using the primer sets ‘1–4' (see Supplementary Table 1 for details) and AmpliTaq Gold (Applied Biosystems).

### Western blot analysis

Cells were snap frozen and lysed with cold RIPA buffer (50 mM Tris-HCl, 150 mM NaCl, 1% NP40, 0.5% sodium deoxycholate, 0.1% SDS (Sigma)) supplemented with Complete EDTA free (Roche) on ice. Lysates were cleared by centrifugation and total protein measured using the BCA assay (Pierce). Equal amounts of protein was loaded and fractionated in SDS–PAGE Tris-Acetate 4–12% gels (Bio-Rad). Proteins were transferred onto Hybond ECL nitrocellulose membranes (Amersham) using wet transfer at 4 °C in wet transfer buffer (25 mM Tris base, 189 mM glycine, 20% methanol). Membranes were washed in PBS-T and blocked with 5% milk in PBS-T (Sigma). Primary and secondary antibody incubations were in 5% milk in PBS-T and membranes were washed in PBS-T. Signals were visualized using ECL (Pierce).

### Flow cytometry

Analysis of DNA content with flow cytometry was done using propidium iodide (PI, Sigma) staining and standard procedures. Samples were analysed on a FACSCalibur (BD Biosciences) flow cytometer. Determination of M-phase content using the MPM-2 antibody (05-368, Millipore) was done according to the manufacturer's protocol using Alexa Fluor (Life technologies) 488 anti-mouse secondary antibody. Polyploidy analysis of HeLa cells was done using a C6 Accuri flow cytometer (BD Biosciences). Cells were fixed, stained with PI and passed through a 40-μm filter to remove cell aggregates and gated using the forward scatter and side scatter filters.

### Antibodies

Primary antibodies used for western blotting were as follows: mouse anti-β-tubulin (T4026, Sigma, 1:6,000), mouse anti-PICH (Ab57434, Abcam, 1:500), goat anti-TopoIIα (sc5347, Santa Cruz, 1:1,000), mouse anti-Actin (A3853, Sigma, 1:6,000), mouse anti-GFP (11814460001, Roche, 1:1,000). Primary antibodies used for indirect immunofluorescence microscopy were as follows: mouse anti-PICH (04-1540, Millipore, 1:100), rabbit anti-PICH (8886S, Cell Signaling, 1:100), guinea pig anti-PICH (in-house, 1:400) mouse anti-β-tubulin (T4026, Sigma, 1:600), rabbit anti-CENPA (a kind gift from Professor Tatsuo Fukagawa published in ref. 30, 1:100), mouse anti-UBF (sc-13125, Santa Cruz, 1:250).

### Indirect immunofluorescence and chromosome spreads from human lymphocytes

DT40 cells were fixed (4% paraformaldehyde in PBS), permeabilized (0.25% Triton-X 100, 1% BSA in PBS), washed and incubated with antibodies on Polysine-coated slides. Slides were mounted in Vectashield with DAPI (Vector laboratories). For immunofluorescence on chromosome spreads, human lymphocytes were treated with 0.1 μg ml^−1^ Karyomax Colcemid (Gibco) for 4 h before being harvested and centrifuged onto glass slides using a Cytospin 4 (Thermo Scientific). The spreads were fixed, incubated with antibodies and mounted in Vectashield with DAPI (Vector laboratories). Alexa Fluor (Life technologies) fluorophore-conjugated secondary antibodies were used to visualize the primary antibodies. Images were captured on either a Nikon Ellipse microscope, an automated upright Olympus BX63 fluorescence microscope or an upright point scanning confocal Axioimager Z2 microscope (Zeiss). Images from confocal microscopy were analysed with ZEN 2010 (Zeiss) otherwise ImageJ was used for image analysis^31^.

### Fluorescently tagged cell lines

Clone *PICH*^*−/−*^ 1 was grown with 4-Tamoxifen to activate the Cre recombinase and to promote excision of the *Bsr* and *Puro* resistance genes. This ‘Loxed' clone was used for all subsequent manipulations. H*PICH* cDNA was PCR amplified with the following primer pair: attB1-PICH and PICH-attB2 (Supplementary Table 1), using pGFP-PICH^8^, pGFP-PICH-T1063A- and pGFP-PICH-K128A as templates. The K128A mutant was generated by QuickChange site-directed mutagenesis (Stratagene). The resulting PCR product was inserted into the pENTR201 Gateway vector (Invitrogen, Life Technologies) via the BP Gateway reaction, and its correct sequence was verified. The *PICH* cDNA insert was subcloned into pDESTzeo-mCherry-C0 via the Gateway LR reaction, to generate fusion constructs tagged with mCherry on the N-terminus. These constructs were linearized then transfected into the *PICH*^*−/−*^ 1 clone by electroporation (Bio-Rad). The cells expressing H2B-GFP were generated by non-targeted transfection transfecting the vector p*H2B*-*GFP*-*Bsr*^*r*^ by electroporation (Bio-Rad) at 950 μF and 250 V and stable clones were selected with 20 μg ml^−1^ Blasticidin (Sigma). The construct used for endogenous tagging of Topo IIα with GFP was a kind gift of Professor William Earnshaw, University of Edinburgh. The construct is a modified version of the p*TOP2A*-Flag construct published previously^32^. The p*TOP2A-GFP* plasmid was linearized with *Not*I and transfected into DT40 cells by electroporation at 25 μF and 550 V and clones were selected with 0.5 μg ml^−1^ Puromycin (Sigma).

### Scoring of chromosomal instability in DT40 cells

For the quantification of micronuclei, cells were treated with 2 μg ml^−1^ Cytochalasin B on poly-L-lysine-coated coverslips (BD Bioscience) for 12 h before being fixed and mounted with Vectashield mounting medium with DAPI (Vector laboratories). Micronuclei in binucleated cells were scored using the micronucleus assay^21^. For the quantification of chromatin bridges and binucleation, cells were harvested following asynchronous growth incubated on Polysine (Thermo Scientific) slides, fixed and mounted with Vectashield mounting medium with DAPI. Slides were scored using a Nikon Ellipse Microscope. Binucleation was defined and scored as in the micronucleus assay.

### Live cell microscopy

Cells were grown in chambered μ-slides (Ibidi) for 15 min in Leibowitz CO_2_-independent media (Gibco), before microscopy at 37 °C using a DeltaVision Elite (Applied Precision) equipped with a × 100∼ objective lens (U-PLAN S-APO, NA 1.4; Olympus), a cooled EMCCD camera (Evolve 512; Photometrics) and a solid-state illumination source (Insight; Applied Precision, Inc). In the experiments with Hoechst staining, 1 μg ml^−1^ Hoechst 33342 (Sigma) was added to the cell suspension 10 min before imaging.

### Establishment of a human *PICH* knockout cell line

A *PICH* knockout cell line was generated by the double nicking strategy, which introduces double strand breaks near the start codon of *PICH* using the D10A mutant Cas9 nickase^33^. Guide RNA sequences (5′-CCGAAGGTTTCCGGAAGCCG-3′ and 5′-GCCTCCATGACCCTCGGATT-3′) were cloned into pSpCas9n(BB)-2A-GFP (PX461) and pSpCas9n(BB)-2A-Puro (PX462) (gifts from Feng Zhang, Addgene plasmid 48140 and 48141), respectively, as suggested by Addgene. These vectors were co-transfected into HeLa cells (obtained from the ATCC) expressing mCherry-H2B for 48 h, followed by collecting GFP-positive cells with a cell sorter. GFP-positive cells were plated on 96-well plates and selected using 0.25 μg ml^−1^ Puromycin at 37 °C for 14–21 days. Viable clones were further cultured in six-well plates. Successful knockout clones were confirmed by western blotting.

### Chromosome spreads from human cells

Mitotic cells were enriched by releasing thymidine-arrested cells into medium containing 0.1 μg ml^−1^ nocodazole for 14 h. ICRF-193 (0.1 μM) was treated together with nocodazole as indicated. Mitotic cells were shaken-off, treated with hypotonic buffer (70 mM KCl) for 15 min, resuspended in Carnoy's fixative (methanol/acetic acid=3:1) and spread onto glass slides. The spreads were mounted in DAPI-containing mounting medium (Vectashield, Vector Labs) and imaged with a Leica DMI6000 inverted microscope and a HCX PLFL × 100/numerical aperture=1.4 oil-immersion objective. Images were acquired and analysed with the MetaMorph software.

### Scoring chromosomal instability in human cells

For the quantification of binucleation and micronucleation in HeLa cells, asynchronous growing cells on coverslips were transiently treated with PKH67 green fluorescent dye for 15 min, fixed and mounted with Vectashield mounting medium with DAPI. Nuclei with diameter smaller than 5 μm were counted as micronuclei. For scoring the percentage of mitotic cells with chromatin bridges, asynchronous growing cells on coverslips were fixed and mounted with Vectashield mounting medium with DAPI. Slides were imaged using a Leica DMI6000 microscope and analysed using the MetaMorph software (Molecular Devices).

### Scoring polyploid cell population in human cells

Asynchronous growing cells were fixed with 70% ethanol, stained with PI, passed through 40 μm filter to remove aggregated cells. Filtered cells were subjected to flow cytometry using the Accuri C6 flow cytometer (BD Biosciences) and analysed using the BD Accuri C6 software.

### Generation of the single catenane substrate

The pSA1101 plasmid for the expression of Tn3 resolvase in BL21(DE3)pLysS bacterial cells was a kind gift from Prof Marshall Stark, University of Glasgow. Purification of Tn3 resolvase was performed under denaturing conditions followed by refolding and renaturation steps of the enzyme, essentially as described previously^34^ with protocol modifications provided by the Stark laboratory. The single catenane substrate was generated by subjecting the pMM5 plasmid to a Tn3 resolvase reaction (25 ng μl^−1^ pMM5, 50 nM Tris-HCl, pH 9.4, 10 mM MgCl_2_, 0.1 mM EDTA, 1/200 Tn3 resolvase prep.) for 3 h. The reaction was stop by addition of 1 M Tris-acetate, pH 7. The pMM5 single catenane was purified first by isopropanol precipitation and then by gel extraction and electro-elution followed by ammonium acetate precipitation The protocol for generating the single catenane substrate was published previously^35^. The pMM5 plasmid was a gift from Professor Stark. Purified PICH protein was a kind gift from Dr Jacqueline H. Enzlin, University of Copenhagen. Pure human Topo IIα was purchased from Inspiralis.

### Decatenation and relaxation assays

For the decatenation assays, the enzymes were pre-incubated on ice for 10 min in a 10-μl reaction mixture containing 20 ng single catenane substrate, 50 mM HEPES, pH 7.4, 100 mM NaCl, 10 mM MgCl_2_, 2 mM dithiothreitol and 50 ng μl^−1^ BSA. ATP was then added to 5 mM final concentration and reactions were incubated at 37 °C for the indicated amount of time. Reactions were stopped by cooling on ice followed by the addition of 0.2% SDS, 100 μg ml^−1^ Proteinase K, 15% sucrose and 25 mg ml^−1^ bromophenol blue and incubated for another 30 min at 37 °C. Reaction products were separated on a 1% agarose gel in the presence of ethidium bromide. Relaxation assays were performed as per the decatenation assays, but in the presence of 100 ng of negatively supercoiled pUC19 as the substrate. Relaxation products were separated on a 1% agarose gel in the absence of ethidium bromide. hTopo IIα and wheat germ Topo I were purchased from Inspiralis and Promega, respectively.

## Additional information

**How to cite this article:** Nielsen, C. F. *et al.* PICH promotes sister chromatid disjunction and co-operates with topoisomerase II in mitosis. *Nat. Commun.* 6:8962 doi: 10.1038/ncomms9962 (2015).

## Supplementary Material

Supplementary Figure and Supplementary Table Supplementary Figures 1-7 and Supplementary Table 1

Supplementary Movie 1 Bulky chromatin bridges in PICH-/- cells. *PICH-/-* cells stably expressing H2B-GFP were followed by time-lapse live cell microscopy after release from nocodazole arrest into medium containing 0.1 μM ICRF-193.

Supplementary Movie 2 UFBs in PICH-/- cells expressing ATPase-dead PICH. *PICH-/-* cells stably expressing H2B-GFP and hPICH-K128A-mCherry were followed by time-lapse live cell microscopy after release from nocodazole arrest into medium containing 0.1 μM ICRF-193.

Supplementary Movie 3 Localization of PICH bodies. 3D projection of hPICH-K128A-bodies in G1 in *PICH-/-* +H2B-GFP (green) +hPICH-K128A-mCherry (red) cells released from nocodazole arrest.
