# Supplementary material for: PICH promotes sister chromatid disjunction and co-operates with topoisomerase II in mitosis
Source: Nat Commun. 2015 Dec 8;6:8962. doi: 10.1038/ncomms9962 (PMC4686863; doi:10.1038/ncomms9962)
Supplement: Supplementary Figure and Supplementary Table — Supplementary Figures 1-7 and Supplementary Table 1 [file ncomms9962-s1.pdf]

Supplementary Figure 1: Polyploidy analysis and drug sensitivity

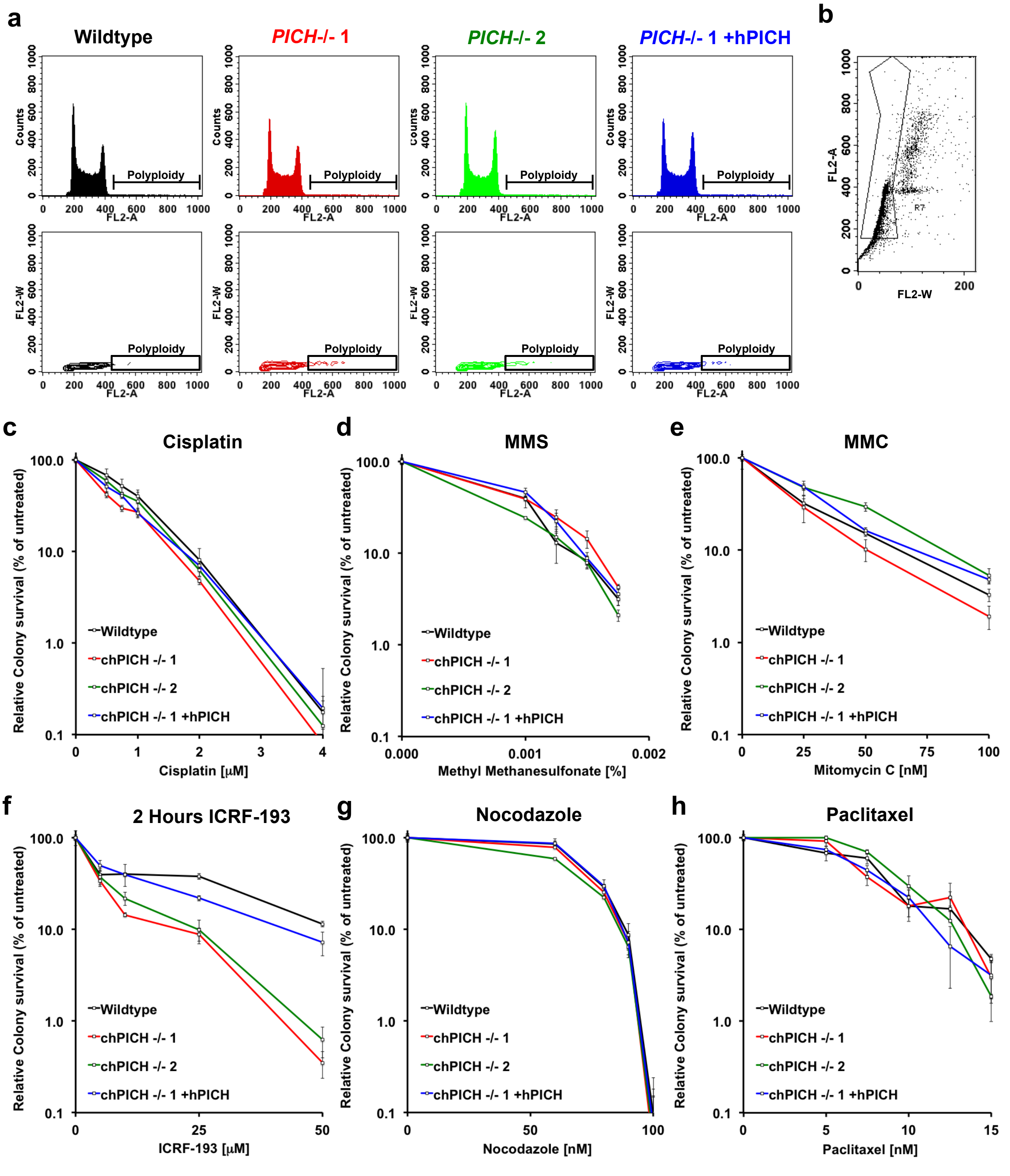

(**a**) Examples of scored FACS profiles from PI-FACS analysis of spontaneous polyploidy. The position of the polyploid cells is indicated. (**b**) The flow cytometry gate used when quantifying polyploidy with PI-FACS, but gating dead cells and aggregates. FL2 area (area of the red fluorescent signal) is plotted in the x-axis and FL2-W (width of the red fluorescent signal) on the Y-axis. Polyploid cells have higher than G2/M FL2-A signal, but retain similar (FL2-W). (**c-i**) Clonogenic survival assays. Cells of the indicated genotypes were exposed continuously to cisplatin (**c**), methylmethane sulfonate (**d**) nocodazole (**h**) or paclitaxel (**i**), or for two hours to mitomycin C (**e**) or ICRF-193 (**f**). Drug concentrations are indicated on the x axes and clonogenic survival relative to plating efficiency on a logarithmic scale is on the y axes. Each data point is the average determinations of at least three independent experiments each done in triplicate (±SD).

Supplementary Figure 2: Mild Topo II inhibition causes *PICH-/-* cells to abort mitosis

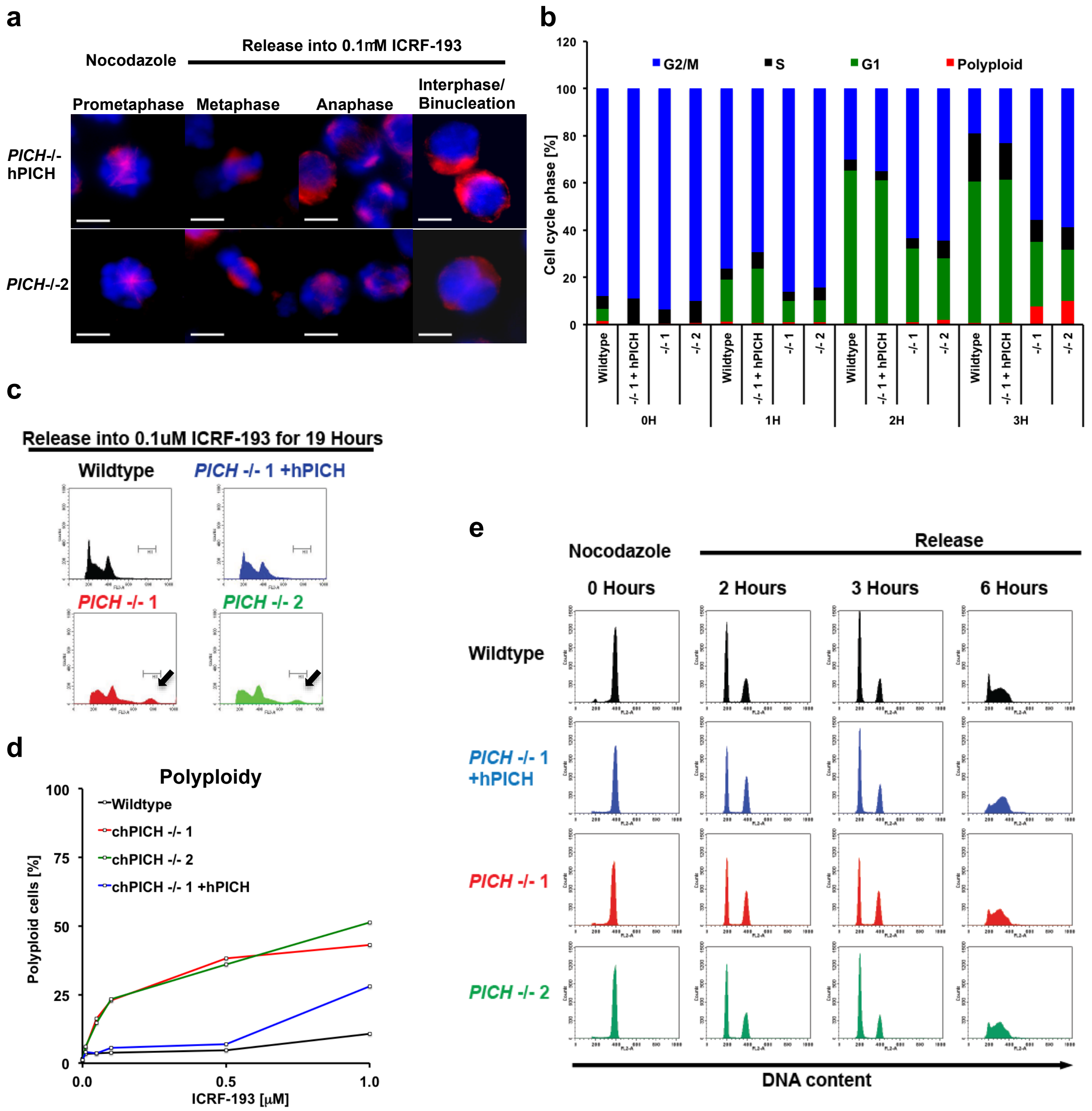

(**a**) Representative examples of mitotic stages and interphase/binucleation in PICH-proficient (*PICH-/-* +hPICH) and -deficient (*PICH-/-*) cells from immunofluorescent staining of β-tubulin (red) combined with DAPI staining of DNA (blue). Scale bar, 5 µm. (**b**) Cell cycle distribution and polyploidy were quantified by PI-FACS/DNA content of the indicated cell lines released from nocodazole treatment into mitosis with 0.1 µM ICRF-193. (**c**) Polyploidy of the indicated cell lines released from nocodazole into medium containing 0.1 µM ICRF-193 for 19 hours and quantified by PI-FACS/DNA content. (**d**) Polyploidy of the indicated cell lines released from nocodazole into medium containing the indicated concentrations of ICRF-193 for 6 hours and quantified by PI-FACS/DNA content. (**e**) PI-FACS profiles of the indicated cell lines released from nocodazole arrest and harvested at the different time points. (**b-e**) Scored events were gated as in Supplementary Fig. 1b.

Supplementary Figure 3: The effect of Replication stress on polyploidy, and phenotypes of *PICH-/-* +hPICH-K128A cells.

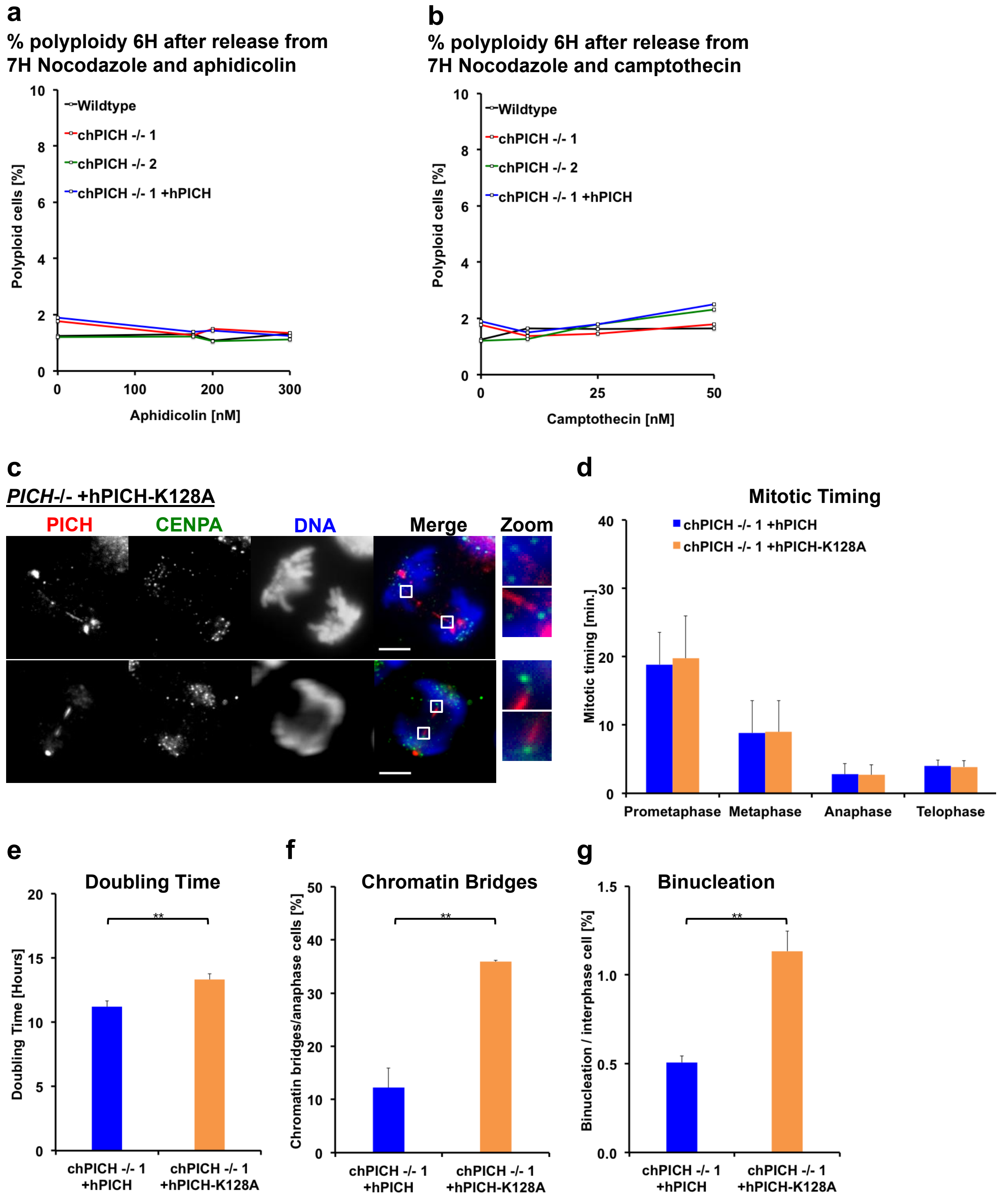

(**a-b**) Cells of the indicated genotypes were treated for seven hours with the indicated concentrations of either aphidicolin (**a**) or camptothecin (**b**) combined with nocodazole to synchronize the cells. Cells were released into drug free medium and harvested for FACS six hours later. Polyploidy was quantified like in Supplementary Fig. 3A-B. (**c**) Microscopy images of late UFBs in *PICH-/-* +hPICH-K128A cells. PICH (red) and CENPA (green). Zoomed images show termini of the UFBs. Scale bars, 5 μm. (**d**) Mitotic timing of *PICH-/-* +H2B-GFP +hPICH (n=48) or PICH-/- +hPICH-K128A (n=48) cells released from nocodazole arrest analysed using time-lapse live cell microscopy. (**e**) Doubling time of *PICH-/-* +hPICH and PICH-/- +hPICH-K128A cells counted using hemocytometer. (**f-g**) Asynchronously growing *PICH-/-* +hPICH and PICH-/- +hPICH-K128A cells were fixed and stained with DAPI. Quantification of spontaneous chromatin bridges (**f**) and binucleation (**g**). (**e-g**) Each data point is an average of at least three independent experiments ±SD. Significance levels were determined using the Students t-test for parametric observations and are indicated as *: P<0.05, **: P<0.01 and ***: P<0.001.

Supplementary Figure 4: PICH bodies in mitosis

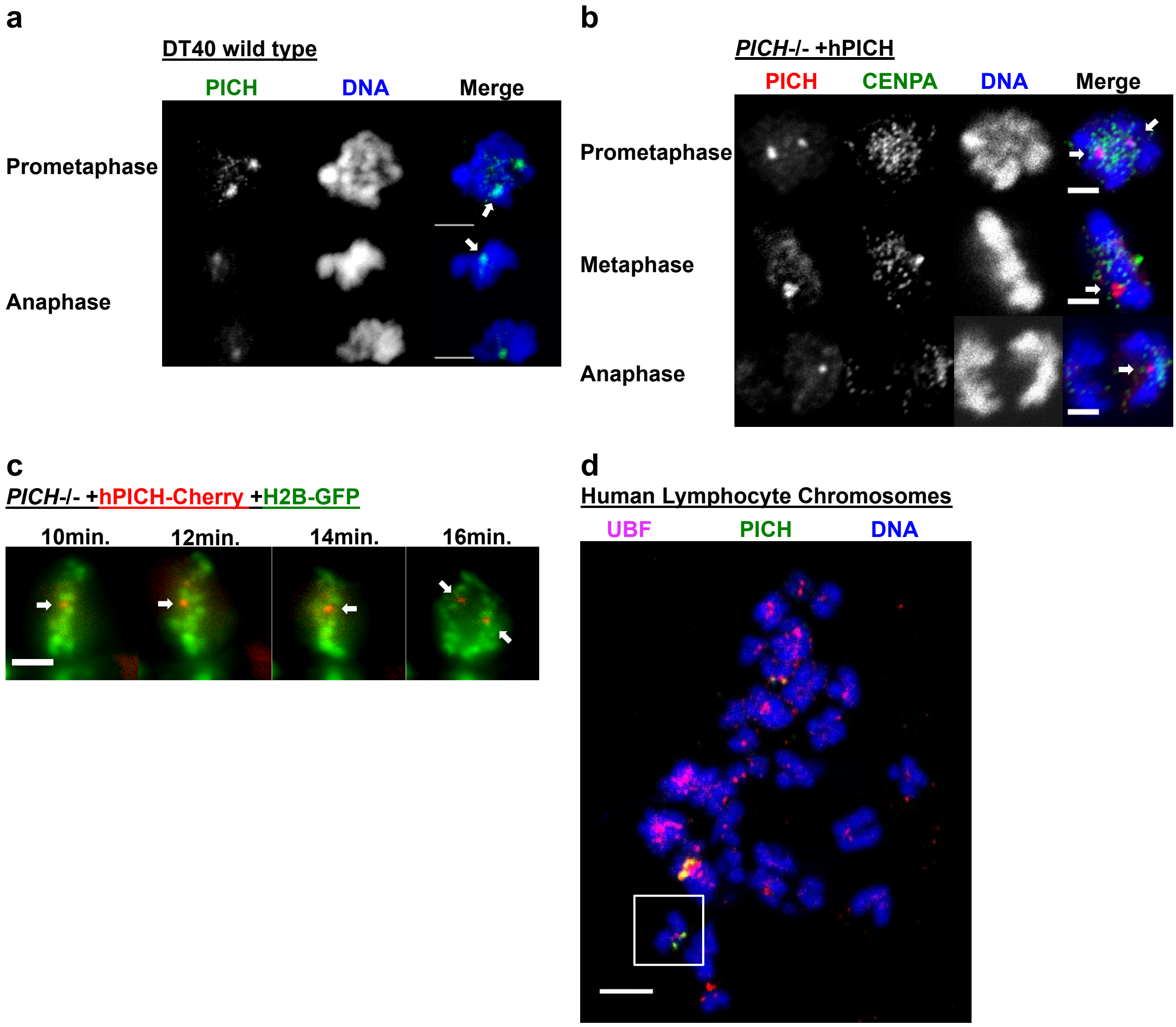

(**a-c**) Cells were arrested with nocodazole and released into drug-free medium. Scale bars, 5 μm. (**a**) Immunofluorescent staining of wild-type chicken *PICH* combined with DAPI staining of DNA in DT40 cells showing PICH bodies in prometaphase and anaphase. White arrows denote PICH bodies. (**b**) Immunofluorescent of PICH (red) and CENPA (green) combined with DAPI staining of DNA in *PICH-/-* +hPICH cells showing PICH bodies in prometaphase, metaphase and anaphase. White arrows denote PICH bodies. The images are single slices from confocal z-stacks. (**c**) Time-lapse microscopy images of *PICH-/-* +hPICH-mCherry (red) +H2B-GFP (green) cells. White arrows denote PICH bodies. (**d**) Confocal imaging of chromosome spreads from human lymphocytes treated with colcemid for 4 hours stained for PICH (green) and UBF (pink). DNA (blue) was staining with DAPI. Red arrows denote UBF-PICH foci and white arrows denote centromeric PICH foci. Scale bar, 5 μm.

Supplementary Figure 5: Confirmation of Topo IIα-GFP expression and Topo IIα-UFBs

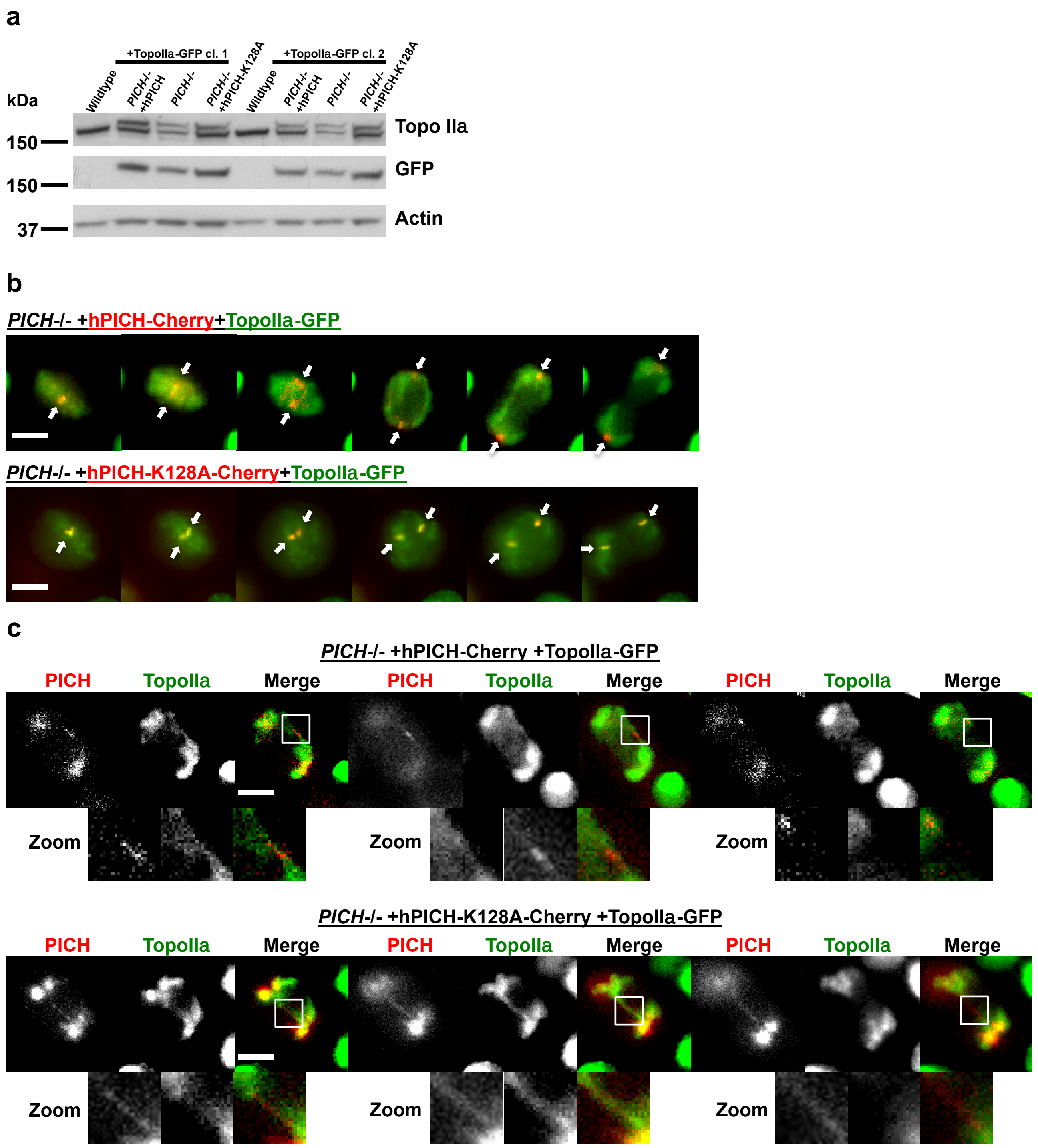

(**a**) Western blot analysis for Topo IIα and GFP to confirm the GFP tagging of the endogenous *TOP2A* locus in whole cell extracts from cells of the indicated genotypes. Untransfected wild-type cells are shown as a control to define the position of the endogenous, untagged Topo IIα protein. Actin was used as a loading control. (**b-c**) Time-lapse microscopy images of *PICH-/-* +Topo IIα-GFP (green) +hPICH-mCherry (red) and +hPICH-K128A-mCherry (red) cells arrested with nocodazole and released into drug-free medium. Scale bars, 5 μm. (**b**) PICH-Topo IIα bodies segregating alongside each sister DNA mass. White arrows denote PICH-TopoIIα bodies. (**c**) The zoomed images highlight UFBs.

Supplementary Figure 6: Phenotypes of *PICH* knockout in human HeLa cells

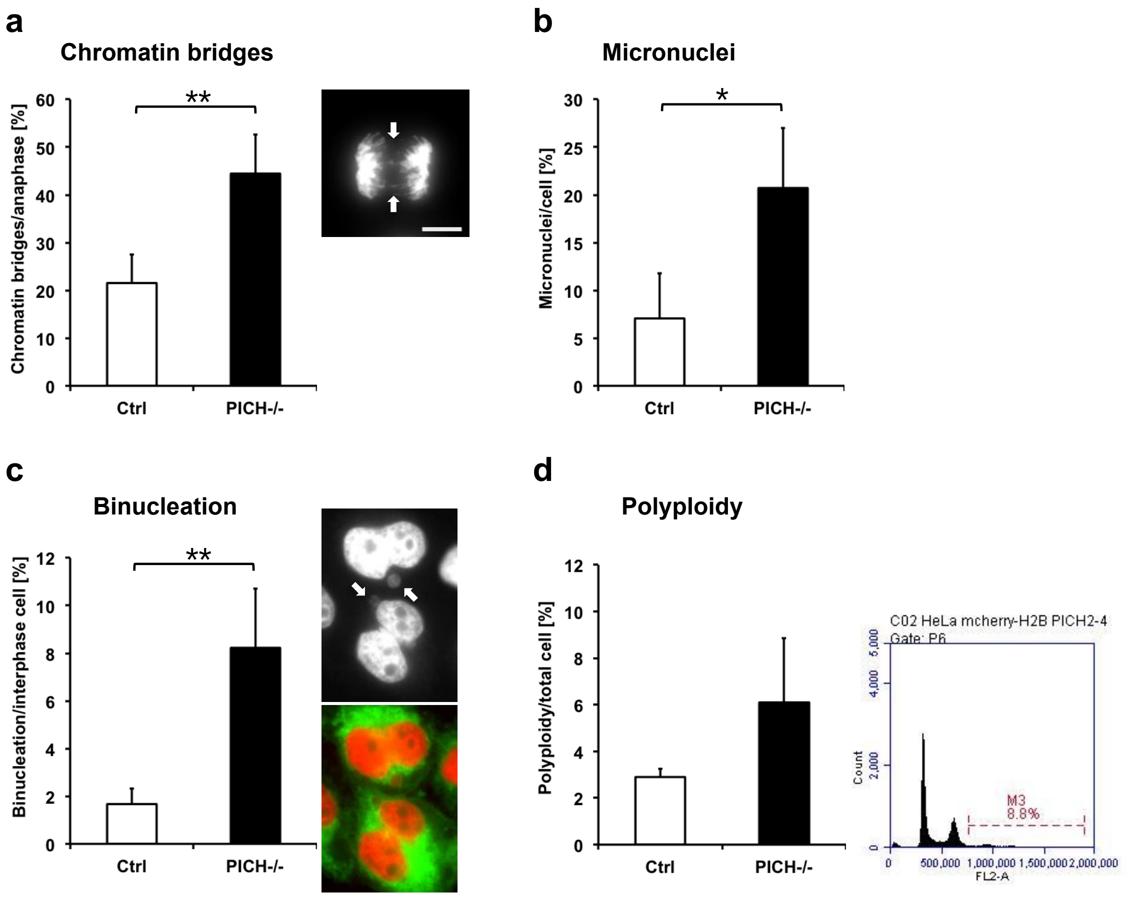

(**a**) Quantification of spontaneous chromatin bridges in HeLa and HeLa *PICH*-/- cells. Cells were fixed and stained with DAPI. An example of an anaphase with chromatin bridges is shown. White arrows denote chromatin bridges. Scale bar, 5 μm. (**b-c**) Cells were fixed and stained with PKH-67 and DAPI. (**b**) Quantification of spontaneous micronucleus formation in HeLa and HeLa *PICH*-/- cells. (**c**) Quantification of spontaneous binucleation in HeLa and HeLa *PICH*-/- cells. An example of binucleated cells with micronuclei is shown. White arrows denote micronuclei. (**d**) Polyploidy of asynchronously growing HeLa and HeLa PICH-/- cells was quantified by FACS as described in the Methods. An example of a scored FACS profile is shown (right).

Supplementary Figure 7: The single catenane substrate and supercoiling assays

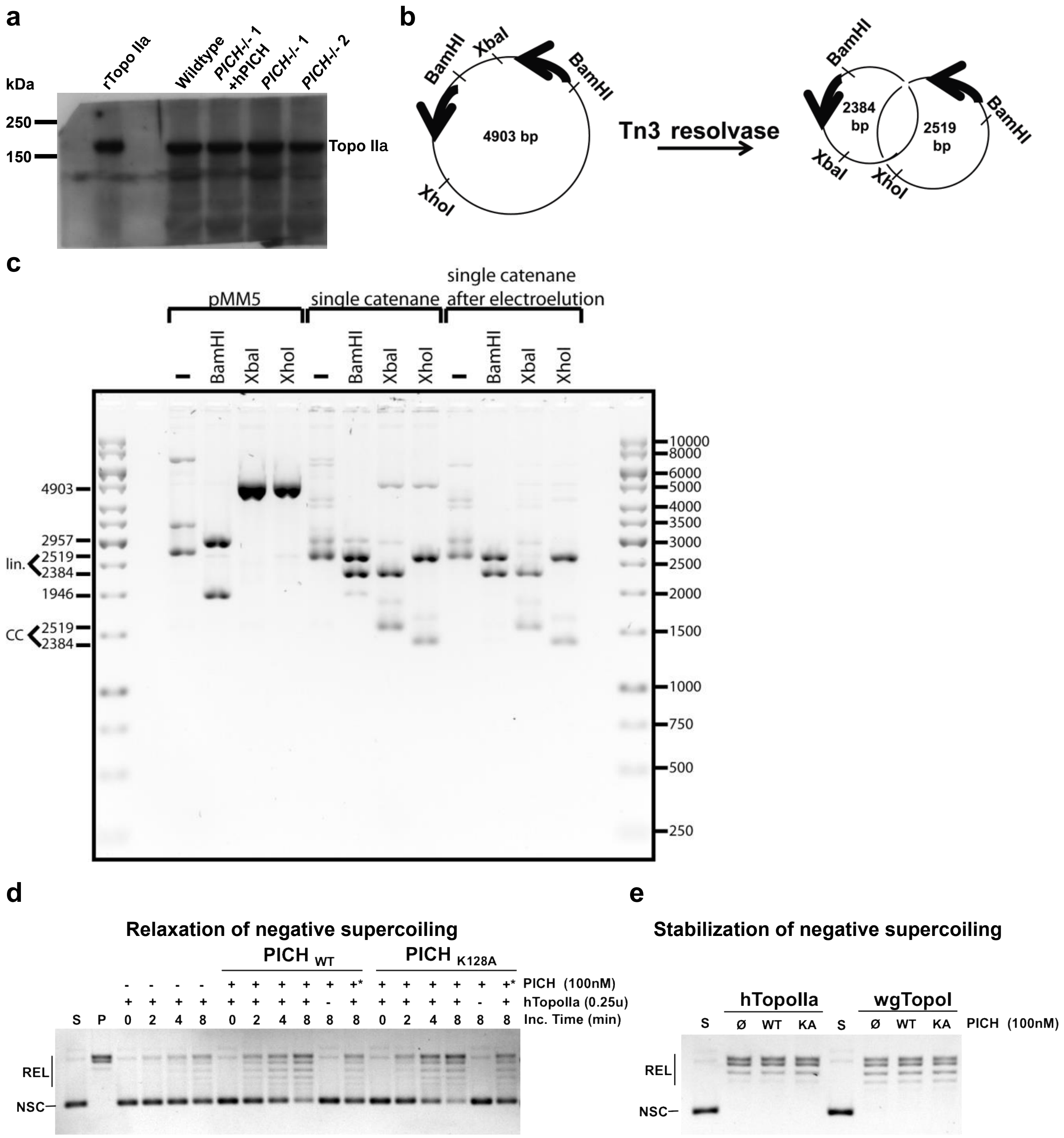

(**a**) Uncropped scan of the Topoisomerase IIα western blot in Figure 9a. Cells were synchronized with nocodazole before harvesting for the preparation of mitotic extracts. (**b**) A Scheme of the resolvase reaction that converts a single circular plasmid into the single catenane substrate (two covalently closed interlinked DNA circles). Tn3 resolvase recognition sites are indicated as black arrows. Restriction sites used to validate the substrate are indicated. (**c**) Validation of formation of the single catenane substrate. Restriction enzyme reactions were carried out to validate and differentiate the single catenane substrate from the initial pMM5 plasmid. (**d**) Relaxation of a negatively supercoiled plasmid. A representative time course assay with the indicated concentrations of hTopo IIα without or with PICH ($PICH_{WT}$) or ATPase-dead PICH ($PICH_{K128A}$). Incubations were terminated at the indicated time points. S: substrate; P: Product (obtained by incubating the substrate in the same conditions but with 2u of Topo IIα); +*: Indicates the lane in which PICH has been heat inactivated at 95°C for 5 min prior to the reaction. NSC: Negatively supercoiled substrate, REL: relaxed products (**e**) Stabilization of supercoiling. Representative relaxation assays incubated for 30 minutes in the presence of 2u of Topo IIα or 2U of Topo I and in the absence (Ø) or in the presence of PICH (WT) or ATPase-dead PICH (KA).

Supplementary Table 1 Primers

Primers	
**Name**	**Sequence 5'-3'**
chPICH-8	CAGCTCAGATGCTACAGGTTCAGC
chPICH-7	AATGGATCCAATGTAAACCTTTGAACAGTCTAATG
chPICH-33	AATGAATTCCAGCATCTTTCTTGACTGTGAG
chPICH-10	CTCCCGGCGCTCCGCACTCAC
1F	CATCCAGCTACTGTTTCTGATG
1R	GTAAGCTTCAAGTTCATTGCCAG
2F	CAGTGCTAATGGAGGTCCTG
2R	CTTGTGATGACAATGCCGTTCT
3F	CAGTGCTAATGGAGGTCCTG
3R	CTTGTGATGACAATGCCGTTCT
4F	TGTCTGGGCCTTCTCAGCAT
4R	TGATGCTTCTCTCCCTGGCA
attB1-PICH	GGGGACAAGTTTGTACAAAAAAGCAGGCTTCGAAGGAGATACCACCATGGAG GCATCCCGAAGGTTTCCGG
PICH-attB2	GCTCTTGACTTTAAGTTTGTATAAGCAACTTAATAACAATGACCCAGCTTTCTT GTACAAAGTGGTCCCC
hPICH-K128A-S	GATGATATGGGATTAGGGGCCACTGTTCAAATCATTGC
